# Immune profiling of SARS-CoV-2 epitopes in asymptomatic and symptomatic pediatric and adult patients

**DOI:** 10.1186/s12967-023-03963-5

**Published:** 2023-02-14

**Authors:** Anna Lucia Tornesello, Chiara Botti, Alberto Micillo, Francesco Labonia, Sergio Arpino, Maria Antonietta Isgrò, Serena Meola, Luigi Russo, Ernesta Cavalcanti, Silvia Sale, Carmine Nicastro, Luigi Atripaldi, Noemy Starita, Andrea Cerasuolo, Ulf Reimer, Pavlo Holenya, Luigi Buonaguro, Franco M. Buonaguro, Maria Lina Tornesello

**Affiliations:** 1 Molecular Biology and Viral Oncology Unit, Istituto Nazionale Tumori IRCCS “Fondazione G. Pascale”, 80131 Naples, Italy; 2 Laboratory of Clinical Pathology, Santobono-Pausilipon Children’s Hospital, 80129 Napoli, Italy; 3 Laboratory Medicine Unit, Istituto Nazionale Tumori IRCCS “Fondazione G. Pascale”, 80131 Naples, Italy; 4 UOC Biochimica Chimica, AORN Ospedali dei Colli P.O. Monaldi, Naples, Italy; 5 JPT Peptide Technologies GmbH, Berlin, Germany; 6 Innovative Immunological Models, Istituto Nazionale Tumori IRCCS “Fondazione G. Pascale”, Via Mariano Semmola, 80131 Naples, Italy

**Keywords:** Severe acute respiratory syndrome coronavirus type 2 (SARS-CoV-2), COVID-19, Peptide microarray, Neutralizing antibodies, Peptide biomarkers, Children SARS-CoV-2 infection

## Abstract

**Background:**

The infection with severe acute respiratory syndrome coronavirus 2 (SARS-CoV-2) has unpredictable manifestations of coronavirus disease (COVID-19) and variable clinical course with some patients being asymptomatic whereas others experiencing severe respiratory distress, or even death. We aimed to evaluate the immunoglobulin G (IgG) response towards linear peptides on a peptide array containing sequences from SARS-CoV-2, Middle East respiratory syndrome-related coronavirus (MERS) and common-cold coronaviruses 229E, OC43, NL63 and HKU1 antigens, in order to identify immunological indicators of disease outcome in SARS-CoV-2 infected patients.

**Methods:**

We included in the study 79 subjects, comprising 19 pediatric and 30 adult SARS-CoV-2 infected patients with increasing disease severity, from mild to critical illness, and 30 uninfected subjects who were vaccinated with one dose of SARS-CoV-2 spike mRNA BNT162b2 vaccine. Serum samples were analyzed by a peptide microarray containing 5828 overlapping 15-mer synthetic peptides corresponding to the full SARS-CoV-2 proteome and selected linear epitopes of spike (S), envelope (E) and membrane (M) glycoproteins as well as nucleoprotein (N) of MERS, SARS and coronaviruses 229E, OC43, NL63 and HKU1 (isolates 1, 2 and 5).

**Results:**

All patients exhibited high IgG reactivity against the central region and C-terminus peptides of both SARS-CoV-2 N and S proteins. Setting the threshold value for serum reactivity above 25,000 units, 100% and 81% of patients with severe disease, 36% and 29% of subjects with mild symptoms, and 8% and 17% of children younger than 8-years reacted against N and S proteins, respectively. Overall, the total number of peptides in the SARS-CoV-2 proteome targeted by serum samples was much higher in children compared to adults. Notably, we revealed a differential antibody response to SARS-CoV-2 peptides of M protein between adults, mainly reacting against the C-terminus epitopes, and children, who were highly responsive to the N-terminus of M protein. In addition, IgG signals against NS7B, NS8 and ORF10 peptides were found elevated mainly among adults with mild (63%) symptoms. Antibodies towards S and N proteins of other coronaviruses (MERS, 229E, OC43, NL63 and HKU1) were detected in all groups without a significant correlation with SARS-CoV-2 antibody levels.

**Conclusions:**

Overall, our results showed that antibodies elicited by specific linear epitopes of SARS-CoV-2 proteome are age dependent and related to COVID-19 clinical severity. Cross-reaction of antibodies to epitopes of other human coronaviruses was evident in all patients with distinct profiles between children and adult patients. Several SARS-CoV-2 peptides identified in this study are of particular interest for the development of vaccines and diagnostic tests to predict the clinical outcome of SARS-CoV-2 infection.

**Supplementary Information:**

The online version contains supplementary material available at 10.1186/s12967-023-03963-5.

## Background

The severe acute respiratory syndrome coronavirus 2 (SARS-CoV-2) has been recognized as the cause of the coronavirus disease 2019 (COVID-19) pandemic which from March 2020 to date has affected over six hundred million people and caused over six million death in the world (covid19.who.int accessed on October 12, 2022). SARS-CoV-2 is an enveloped virus, which belongs to the betacoronavirus genus also containing SARS-CoV and Middle East Respiratory Syndrome virus (MERS-CoV), previously involved in SARS and MERS major epidemics, respectively [1–3]. Although SARS-CoV-2 has a lower death rate than SARS-CoV and MERS-CoV, its high infectivity and easy transmission are causing enormous health problems as well as socio-economic distress in the last three years [4, 5]. Other coronaviruses infecting humans are 229E, OC43, NL63 and HKU1, which cause up to 30% of mild respiratory illnesses and common cold each year [6].

The 30 kilobases single-stranded SARS-CoV-2 RNA genome contains 14 open reading frames (ORFs) encoding 29 structural, non-structural, and accessory proteins [7]. Four structural proteins, namely spike (S), membrane (M) and small envelope (E) glycoproteins as well as nucleocapsid (N) protein that serve as key factors for SARS-CoV-2 binding to target cells, the release of the viral genome and packaging of viral RNA inside the virions [7]. Sixteen non-structural proteins produced by proteolytic digestion of polyproteins 1a (pp1a) and 1ab (pp1ab) with main (Mpro) and papain-like (PLpro) proteases, are required for virus replication and RNA transcription [8]. Accessory proteins encoded by ORF3a, ORF3b, ORF6, ORF7a, ORF7b, ORF8b, ORF9b, ORF9c and ORF10 are important regulators of the virus life cycle but, with the exception of ORF3a and ORF7a proteins, they are not incorporated in mature virions [9, 10].

The antibody response against SARS-CoV-2 proteome is mainly directed against S glycoprotein and N protein [11]. The S glycoprotein forms homotrimers on the virus envelope that mediate the attachment of SARS-CoV-2 particles to target cells through recognition of human angiotensin converting enzyme 2 (ACE2) [12]. The cleavage of full-length S protein by furin-like proteases produces S1 subunit, interacting with ACE2 receptor, and S2 subunit facilitating the fusion of the virus envelope with the host membrane [13]. The wide cell tropism of SARS-CoV-2 is determined by the high expression of ACE2 in type II alveolar cells of the lower respiratory tract and epithelial cells of the upper esophagus, small intestine, colon, kidneys, heart and liver [14]. The S1 receptor binding domain (RBD), is composed of five antiparallel β sheets (β1, β2, β3, β4 and β7) connected by short helices and loops. S glycoprotein residues that make contact with ACE2 receptor form a protein motif composed of short β5 and β6 as well as α4 and α5 helices and loops located between the β4 and β7 strands [15]. The RBD region, identified as the immunodominant domain of S protein, elicits SARS-CoV-2 neutralizing antibodies. Notably, the levels of anti-RBD immunoglobulin G (IgG) highly correlate with the neutralizing activity of patient sera [16, 17]. In addition, S1 domains other than RBD have reported to stimulate strong antibody response with some epitopes eliciting neutralizing activity [18, 19]. S1 protein and RBD region are currently the most focused target for the development of vaccines against SARS-CoV-2.

The N protein is a heavily phosphorylated internal viral antigen, which is mainly involved in viral RNA encapsulation. The mechanism of antibody production against N protein is not well known. Recent studies showed that the cytosolic antibody receptor TRIM21 is able to bind antibody-antigen complexes and to enhance their degradation through the proteasome, thus facilitating the binding of peptides to MHC molecules [20]. A similar mechanism has been proposed for the generation of antibodies directed against the N antigens of SARS-CoV-2 [20].

Clinical manifestations of SARS-CoV-2 infection in adults differ from that in children [21]. Adults frequently develop respiratory symptoms, which in the most severe form can progress to acute respiratory distress syndrome (ARDS), while children are largely spared from respiratory disease remaining asymptomatic, although in few cases they can develop a life-threatening multisystem inflammatory syndrome (MIS) [22].

The immune response against SARS-CoV-2 plays a critical role in dictating clinical outcome in both adults and children [23].

In this retrospective study, we performed a screening of anti-coronaviruses antibody levels in the sera of adults and children with asymptomatic, mild or severe SARS-CoV-2 infection as well as not-infected-vaccinated subjects with mRNA BNT162b2 vaccine against SARS-CoV-2 spike. We analyzed IgG reactivity towards peptides derived from the full SARS-CoV-2 proteome as well as from S, E, N and M proteins of coronaviruses SARS-CoV, MERS-CoV, and common cold human coronaviruses 229E, OC43, NL63 and HKU1 (isolates 1, 2 and 5). The antibody profile allowed to identify specific epitopes in the SARS-CoV-2 proteins, which were differentially recognized by sera of SARS-CoV-2 infected children and adult patients with diverse clinical symptoms.

## Methods

### Patients and biological samples

We conducted a retrospective study, which included 79 subjects, comprising 19 SARS-CoV-2 infected pediatric patients (aged 0–12 years), 14 and 16 SARS-COV-2 infected adults with asymptomatic/mild/moderate symptoms and severe/critical symptoms, respectively, as well as 30 non-infected subjects who received the first dose of BNT162b2 mRNA vaccine between 15 and 20 days before blood sample collection.

Serum samples from pediatric patients, who were hospitalized for pathologies other than COVID-19 and tested positive for SARS-CoV-2 on their hospital admission, were collected at the A.O.R.N. Santobono-Pausilipon. Aliquots of sera from adult patients with asymptomatic/mild/moderate symptoms and vaccinated subjects were collected during the health surveillance program for healthcare workers at the Istituto Nazionale Tumori IRCCS Fondazione G. Pascale. Serum samples from patients with severe/critical symptoms were collected during hospitalization at A.O.R.N. Ospedali dei Colli, Napoli.

On the basis of World Health Organization (WHO) guidelines, patients were classified as asymptomatic if they had no clinical signs or symptoms throughout the course of their infection, with mild/moderate illness if they suffered from cold-like symptoms, dyspnea, anosmia or ageusia, and severe/critical disease if they were hospitalized needing oxygen support or intensive care [https://www.who.int/publications/i/item/WHO-2019-nCoV-clinical-2021-2]. Age, gender, severity of symptoms, clinical data and laboratory parameters, when available, were obtained from medical records of patients. SARS-CoV-2 infection was determined by quantitative real-time reverse transcription-polymerase chain reaction (qRT-PCR) assay performed on nasopharyngeal fluids. Serum samples were obtained from the whole blood by centrifugation at 1200 g for 15 min and then stored at 80 °C. Parents of pediatric patients signed a specific consent for data treatment, approved by the Santobono hospital.

Blood biomarkers, including D-dimer, were tested with regulatory agency-approved and commercially available kits according to the manufacturers’ instructions.

The study was approved by the Institutional Scientific Board and by the Ethics Committee of the Istituto Nazionale Tumori IRCCS Fondazione G. Pascale (number 34/21) and is in accordance with the principles of the Declaration of Helsinki. Signed consent was obtained from health workers participants and from hospitalized patients.

### Peptide microarray

Peptide libraries were manufactured by JPT Technologies GmbH (Berlin) based on the genomic sequence of the original SARS-CoV-2 Wuhan strain (NC_045512.2 and Uniprot e.g. for SPIKE it is P0DTC2). The RepliTope™ Antigen Collection Pan-Coronavirus microarrays (JPT Product Code: RT-HD-CoV2; microarray series #3364) contains 5,828 peptides spanning the full proteome of SARS-CoV-2 and S, E, N and M glycoproteins as well as N protein of SARS-CoV, MERS and coronaviruses 229E, OC43, NL63 and HKU1 (isolates 1, 2 and 5). The number of peptides generated for each viral protein is reported in Supplementary Additional file 1: Table S1. The peptides were 15-amino acids long, overlapping in most cases by 11 residues, and covered the full length of each, above described, viral protein. Mouse and human full-length IgG were co-immobilized on the peptide microarray slides and used as qualitative controls in each assay. Twenty-one 15-mer amino acid sequences were included in each array as internal controls. Peptide microarrays were prepared as described earlier [24]. Briefly, peptides were synthesized with a fully automated system by using SPOT synthesis technology [25] and were subsequently spotted in triplicate and immobilized onto glass slides.

### Microarray assay conditions

Sera were probed on peptide arrays as follows: (1) Individual samples from 12 children up to 7 years old, pooled sera from four 8–9 years old (ped 8–9) and from three 10–12 years old (ped 10–12) children; (2) individual sera from 14 adult patients with asymptomatic/mild/moderate symptoms; (3) individual sera from 16 hospitalized patients with severe/critical symptoms; (4) three pools of samples (VAX-B1, VAX-B2, VAX-B3) each containing sera from ten uninfected individuals who received the first dose of anti-SARS-CoV-2 BNT162b2 mRNA vaccine. Sera samples were diluted 1:200 in T20 Superblock assay buffer (Thermo Scientific™, Waltham, USA), loaded on microarray slides and incubated for 2 h at 30 °C using TECAN HS4800 (Tecan Trading AG, Männedorf, Switzerland) microarray processing station. After sample incubation the slides were washed with 50 mM TBS-buffer containing 0.1% Tween 20 at pH 7.2 and 3 mM SSC buffer at pH 7.0 and then incubated for 45 min at 30 °C with the anti-human IgG fluorescent labeled Alexa Fluor 647 antibody (Jackson Immunoresearch, Cambridge UK), at the concentration of 0.1 µg/ml. After washing with TBS buffer and drying, the slides were scanned with a high-resolution Genepix 4300A SL50 microarray scanner (Molecular Devices LLC, San Josè, USA) at 635 nm. The mean pixel intensity value was calculated for each peptide. All reactions were completed in three days. Simultaneous incubations with the secondary antibody without serum samples were performed to assess nonspecific binding to each synthetic peptide.

### Statistical analysis

The mean value of three replicates on the microarray (MMC2) was calculated for each peptide. If the coefficient of variation (standard deviation divided by the mean value) was greater than 0.5, the mean of the two closest values (MC2) was assigned to MMC2. Heatmaps of all analyzed samples against SARS-CoV-2 N, S and M proteins as well as against only the N protein of 229E, OC43, MERS and SARS-CoV are reported in the Additional file 6: Figure S1. Fluorescence intensities are shown in a color-coded manner from white (no binding) over yellow (medium binding) to red (strong binding). Data analysis and heatmap generation was performed by using the statistical computing and graphics software R (Version 4.0.2, www.r-project.org). Differences in signal intensities between two sample groups (i.e. adults versus children) were analyzed by the Wilcoxon Rank Sum test (R package stats). The ROC analysis was performed by using the R package ROCR. Peptide signals with significant difference between the groups was based on the accuracy value obtained with the formula “(true positive + true negative)/total number of observations”. To obtain a statistically significant difference for the immune response level between groups a threshold above 25′000 was introduced instead of the above 10′000 initial threshold.

Further data analyses have been done using GraphPad Prism (GraphPad Software, version 8.0.0).

## Results

In this study, we included 19 pediatric and 30 adult patients with active or recent SARS-CoV-2 infection and 30 non-infected adults who received the first dose of a SARS-CoV-2 vaccine. All pediatric patients were less than 12 years old and their serum samples were obtained after a positive SARS-CoV-2 rapid antigenic assay at the hospital admittance followed by a confirmation test by real-time PCR. Twelve children were under the age of 8 and their sera were tested individually. In addition, two pools of sera obtained from four children aged 8–9 and three children aged 10–12 were also analyzed. All children were admitted to the hospital for pathologies other than COVID-19. Sera samples from SARS-CoV-2 infected adults were collected approximately 30 days post-diagnosis in the group with asymptomatic/mild/moderate disease, and between 5–30 days post-symptom onset in the group with severe/critical symptoms. The age, gender and clinical status of patients is reported in Table 1.

**Table 1:** Patients with SARS-CoV-2 infection comprised children, adults with asymptomatic/mild/ moderate symptoms and adults with severe/critical symptoms (hospitalized in sub-intensive or intensive care unit)

Characteristic	Pediatric° n = 19 (%)	Adults (Asymptomatic/mild/ moderate) n = 14 (%)	Adults Severe/critical symptoms N = 16 (%)	P value^
Age				
≤ 12 years	19	/	/	
30–45 years	/	4 (28.6)	3* (18.7)	0.533
> 45 years	/	10 (71.4)	13** (81.3)	
Sex				0.157
Male	11 (57.9)	8 (57.1)	13 (81.3)	
Female	8 (42.1)	6 (42.9)	3 (18.7)	

°All pediatric patients were affected by pathologies other than COVID-19

^P value was calculated to evaluate differences between the adult groups

^*^The group comprises one female and 2 males

^**^The group comprises 2 females and 11 males

The male gender predominated among adult patients with severe symptoms (81.3%) and was slightly higher than female gender among subjects with asymptomatic/mild/moderate symptoms (57.1%). In addition, most adults were over 45 years of age, regardless of the severity of their symptoms (71.4% and 81.3%, respectively). The mild/moderate symptoms group included three asymptomatic subjects as well as three and eight subjects with mild and moderate symptoms, respectively. The group of patients with severe/critical symptoms included 3 patients in pre-intensive care and 13 patients in intensive care. In addition, three pools each containing sera from 10 uninfected vaccinated subjects for a total of 30 samples were also analyzed.

### Analysis of antibody levels against SARS-CoV-2 proteome

**Fig. 1:**
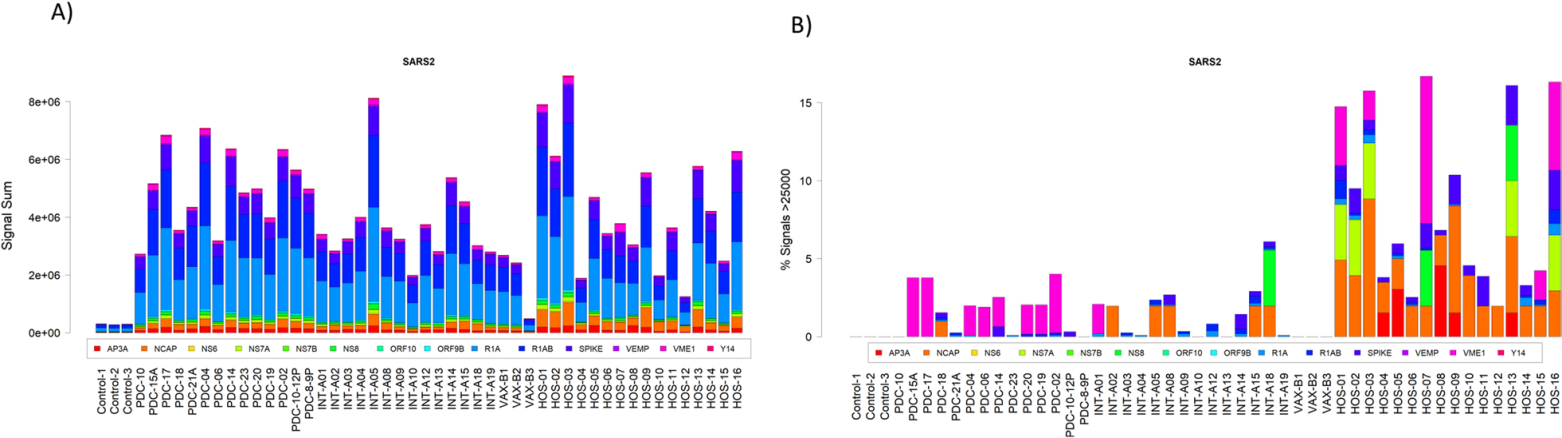
Barplot **A** showing the sum of signals for each protein. Samples labeled as control are samples incubated with the secondary antibody only (n = 3), the PDC are from SARS-CoV-2 positive children, INT indicates adults with mild/moderate symptoms and HOS identifies hospitalized patients with severe symptoms. Samples labeled as VAX-B1, VAX-B2 and VAX-B3 are each a pool of sera from 10 vaccinated subjects. Barplot **B** showing the fraction of signals above the threshold of 25'000. Samples are labelled as in **A**

To evaluate the IgG antibody response against SARS-CoV-2 and human coronaviruses SARS-CoV, MERS, 229E, OC43, NL63 and HKU1 (isolates 1, 2 and 5), a peptide microarray covering the full SARS-CoV-2 proteome and S, E, N and M proteins of other human coronaviruses was used for the serological assays [26]. Serum samples from all SARS-CoV-2 infected patients showed high reactivity against numerous peptides derived from several proteins of SARS-CoV-2 proteome (Fig. 1A). In particular, we observed strongest fluorescent signals against peptides derived from N, S, R1A and R1AB proteins in all samples from SARS-CoV-2 infected subjects. Figure 1A shows for each enrolled subject the sum of fluorescent signals obtained against all peptides for each SARS-CoV-2 protein. Several samples in the control group and in the vaccinated non-infected individuals (VAX-B1, VAX-B2, and VAX-B3) showed some degree of reactivity, which may be due to non-specific antibody binding or to cross-reactive immune response against antigens common to other coronavirus strains.

The IgG responses against each SARS-CoV-2 and other human coronaviruses peptides varied between individuals and patient groups and was considerably higher among those with severe symptoms. Overall, the color-coded heatmaps, reproducing the signal intensity for each peptide, showed an increased reactivity towards those corresponding to the C-terminus and central region of either N or S proteins, the C-terminus of ORF3A and the central region of NS7A in all analyzed samples (Additional file 6: Figure S1). Interestingly, samples from children less than 8 years old reacted mostly against the N-terminus peptides of the M protein while adults mainly reacted against the C-terminus.

To identify the immunodominant epitopes, we set threshold values above 25′000 and observed that all sera samples (100%, 16 out of 16) obtained from severe symptoms patients, 36% (5 out of 14) of individuals with mild symptoms, 8% (1 out of 12) of 8-years old and none of 8–12 years old children reacted against specific peptides of SARS-CoV-2 N protein (Fig. 1B). Similarly, sera from most patients with severe disease (81%) and several of those with mild symptoms (29%) as well as children younger than 8 years (17%) strongly reacted against the S protein domains (Additional file 7: Figure S2-A). On the other hand, the percentage of samples with high signal intensity against SARS-CoV-2 M peptides was very high among children below 8-years (67%, 8 out of 12), high among severe symptoms patients (31%, 5 out of 16) and low in the remaining groups (≤ 7%) (Additional file 7: Figure S2-B).

To identify the most immunogenic peptides in each patient group, we compared the antibody signal intensities of mild versus severe symptoms patients, children versus mild symptoms subjects as well as children versus severe symptoms patients (Table 2). By setting the p value to < 0.01 we identified 10 peptides, differentiating adults with mild/moderate symptoms from those with severe symptoms, and 30 peptides distinguishing children from adults with mild/moderate or severe symptoms (Table 2).

**Table 2:** Linear peptides of SARS-CoV-2 proteome differentially recognized by IgG of adults and children

Peptide	Organism	Protein	Sequence*	**p-value**	**Comparison groups**	**AUC**
Adults						
NCAP_SARS2_0157-0171	SARS-CoV-2	N	IVLQ**LPQGTTLPKGF**	9.9X10^−3^	Mild/Sev	0.773
NCAP_SARS2_0161-0175	SARS-CoV-2	N	**LPQGTTLPKGF**YAEG	4.9X10^−3^	Mild/Sev	0.796
NCAP_SARS2_0221-0235	SARS-CoV-2	N	LLLLDRLNQLESKMS	1.6X10^−5^	Mild/Sev	0.924
NCAP_SARS2_0393-0407	SARS-CoV-2	N	TLLPAADLDDFSKQL	1X10^−3^	Mild/Sev	0.84
SPIKE_SARS2_0557-0571	SARS-CoV-2	S	KKFLPFQQFGRDIAD	8.6X10^−3^	Mild/Sev	0.778
SPIKE_SARS2_0785-0799	SARS-CoV-2	S	VKQI**YKTPPIKDFGG**	1X10^−4^	Mild/Sev	0.889
SPIKE_SARS2_0789-0803	SARS-CoV-2	S	**YKTPPIKDFGG**FNFS	2X10^−3^	Mild/Sev	0.822
SPIKE_SARS2_1145-1159	SARS-CoV-2	S	LDSFKEELDKYFKNH	1.2X10^−3^	Mild/Sev	0.836
R1A_R1AB_SARS2_0249-0263	SARS-CoV-2	1A-1AB	YELQTPFEIKLAKKF	4.8X10^−3^	Asym/mild	0.926
R1AB_SARS2_6073-6087	SARS-CoV-2	1AB	HLIPLMYKGLPWNVV	5.7X10^−3^	Mild/Sev	0.791
Children						
NCAP_SARS2_0109-0123	SARS-CoV-2	N	YFYYLGTGPEAGLPY	9.1X10^−3^	Ped/Mild	0.2
NS7A_SARS2_0009-0023	SARS-CoV-2	NS7A	LITLATCELYHYQEC	3.2X10^−3^	Ped/Mild	0.18
NS8_SARS2_0033-0047	SARS-CoV-2	NS8	VDDPCPIHFYSKWYI	5.9X10^−3^	Ped/Mild	0.2
R1A_R1AB_SARS2_2001-2015	SARS-CoV-2	1A-1B	ATYKPNTWCIRCLWS	3X10^−4^	Ped/Mild	0.12
R1A_R1AB_SARS2_2157-2171	SARS-CoV-2	1A-1B	VTRCLNRVCTNYMPY	1.3X10^−5^ 4.3X10^−3^	Ped/Mild Ped/Sev	0.07 0.2
R1A_R1AB_SARS2_3005-3019	SARS-CoV-2	1A-1B	VLNNDYYRSLPGVFC	5X10^−4^	Ped/Mild	0.13
R1A_R1AB_SARS2_3469-3483	SARS-CoV-2	1A-1B	AWLYAAVINGDRWFL	5.1X10^−3^	Ped/Mild	0.2
R1A_R1AB_SARS2_4269-4283	SARS-CoV-2	1A-1B	FCAFAVDAAKAYKDY	3.7X10^−3^	Ped/Mild	0.19
R1AB_SARS2_4541-4555	SARS-CoV-2	1A-1B	YNCCDDDYFNKKDWY	9.1X10^−3^	Ped/Mild	0.2
R1AB_SARS2_4653-4667	SARS-CoV-2	1A-1B	LTKPYIKWDLLKYDF	1X10^−4^	Ped/Mild	0.1
R1AB_SARS2_4673-4687	SARS-CoV-2	1A-1B	KLFDRYFKYWDQTYH	1.6X10^−3^	Ped/Mild	0.16
R1AB_SARS2_4725-4739	SARS-CoV-2	1A-1B	IFVD**GVPFVVSTGYH**	6.8X10^−3^	Ped/Mild	0.2
R1AB_SARS2_4729-4743	SARS-CoV-2	1A-1B	**GVPFVVSTGYH**FREL	7.9X10^−3^	Ped/Mild Ped/Sev	0.2 0.16
R1AB_SARS2_4809-4823	SARS-CoV-2	1A-1B	KDFYDFAVSKGFFKE	5.9X10^−3^	Ped/Mild	0.2
R1AB_SARS2_4845-4859	SARS-CoV-2	1A-1B	YDYYRYNLPTMCDIR	6X10^−4^	Ped/Mild	0.14
R1AB_SARS2_5273-5287	SARS-CoV-2	1A-1B	FHLYLQYIRKLHDEL	5X10^−4^	Ped/Mild	0.13
R1AB_SARS2_5829-5843	SARS-CoV-2	1A-1B	AWRKAVFISPYNSQN	6.8X10^−3^	Ped/Sev	0.27
R1AB_SARS2_6073-6087	SARS-CoV-2	1A-1B	HLIPLMYKGLPWNVV	4X10^−4^	Ped/Mild	0.133
R1AB_SARS2_6153-6167	SARS-CoV-2	1A-1B	HHSIGFDYVYNPFMI	2.7X10^−3^	Ped/Mild	0.18
R1AB_SARS2_6681-6695	SARS-CoV-2	1A-1B	GYAFEHIVYGDFSHS	7.9X10^−3^	Ped/Mild	0.2
R1AB_SARS2_6973-6987	SARS-CoV-2	1A-1B	SWNADLYKLMGHFAW	1.3X10^−3^	Ped/Mild	0.16
SPIKE_SARS2_0133-0147	SARS-CoV-2	S	FQFCNDPFLGVYYHK	8X10^−4^	Ped/Mild	0.148
SPIKE_SARS2_0265-0279	SARS-CoV-2	S	YYVGYLQPRTFLLKY	7.9X10^−3^	Ped/Sev	0.214
SPIKE_SARS2_0325-0339	SARS-CoV-2	S	SIVRFPNITNLCPFG	1.6X10^−3^ 2.7X10^−3^	Ped/Mild Ped/Sev	0.167 0.181
SPIKE_SARS2_0553-0567	SARS-CoV-2	S	TESNKKFLPFQQFGR	3.7X10^−3^	Ped/Mild	0.19
SPIKE_SARS2_0557-0571	SARS-CoV-2	S	KKFLPFQQFGRDIAD	3.2X10^−3^	Ped/Mild	0.186
SPIKE_SARS2_0785-0799	SARS-CoV-2	S	VKQIYKTPPIKDFGG	2.7X10^−3^	Ped/Mild	0.181
SPIKE_SARS2_1201-1215	SARS-CoV-2	S	QELG**KYEQYIKWPWY**	9X10^−4^	Ped/Mild	0.15
SPIKE_SARS2_1205-1219	SARS-CoV-2	S	**KYEQYIKWPWY**IWLG	3.7X10^−3^	Ped/Mild	0.19
VME1_SARS2_0005-0019	SARS-CoV-2	M	NGTITVEELKKLLEQ	7.9X10^−3^	Ped/Mild	0.2

^*^The amino acids highlighted in bold indicate overlapping sequences between different peptides in each protein

The sera of patients with severe symptoms were highly reactive against all SARS-CoV-2 peptides compared to individuals with mild or moderate symptoms (Additional file 8: Figure S3-A). In particular, two epitopes of the N protein, aa 157–171 and aa 161–175, showed the highest signals (> 18′000 Units) when probed with the sera of patients with severe symptoms. High IgG reactivity against these two peptides was also observed with sera samples of five subjects presenting moderate symptoms. Moreover, both children or adult patients with severe symptoms, but not those from other subjects, developed antibodies with high reactivity against the SARS-CoV-2 S aa 557–571 peptide (Additional file 8: Figure S3-AB)( Additional file 9: Figure S4). The sera of children, similarly to adult patients with severe disease, showed a significantly stronger reaction against 25 peptides, mainly of the R1AB and S proteins, compared to adults with mild or moderate symptoms (Table 2). In addition, children and adult patients with severe symptoms showed a similar high antibody response against two domains, namely the aa 5–19 of the M protein as well as the aa 553–567 of the S protein, which was not observed in the other patient group. Importantly, high IgG levels against amino acids 785–799 of S protein were specifically detected in patients with severe symptoms but not in other patient groups (Additional file 8: Figure S3-B)( Additional file 9: Figure S4).

### Analysis of antibody levels against SARS-CoV, MERS-CoV, OC43, 229E, HKU1 and NL63

**Fig. 2:**
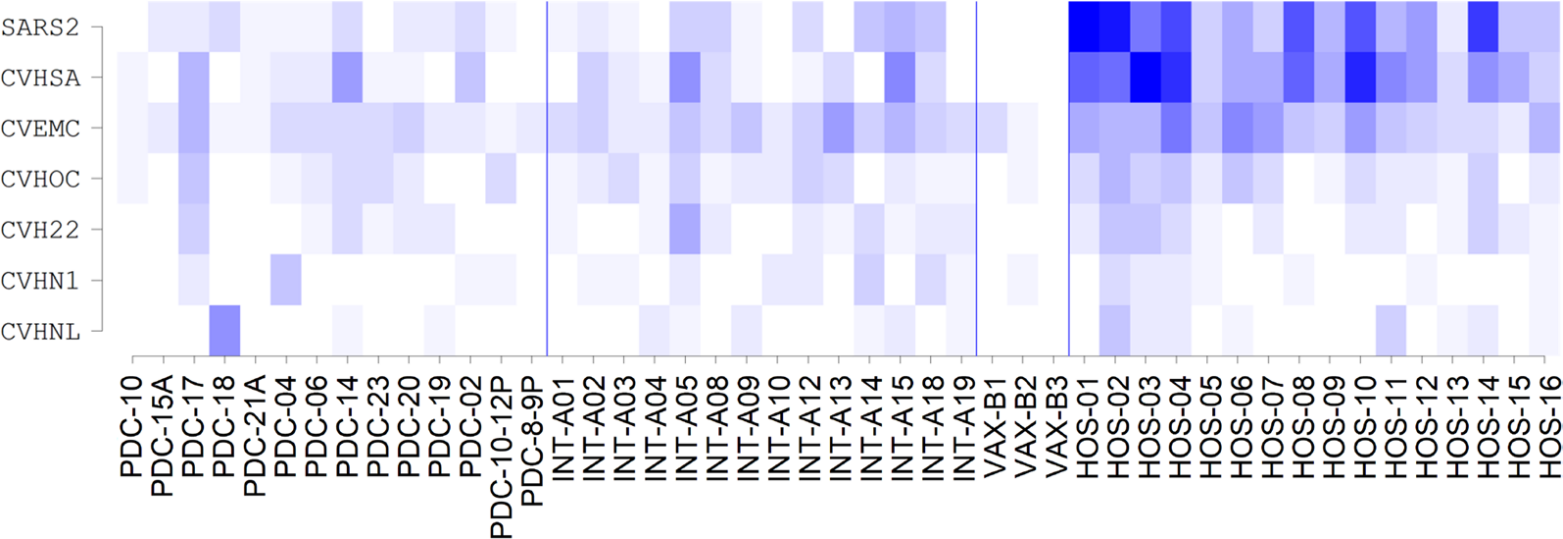
The heatmap depicts the humoral response of COVID-19 patients to the epitopes of N and S proteins of human coronaviruses with a signal threshold above 25'000. Samples labeled as PDC are children below 8 years of age, PDC.8-9p and PDC.10-12p are pools of sera from children in the range 8–9 and 10–12 years of age, respectively. Samples labeled as INT are from patients with mild or moderate symptoms, HOS are from patients with severe symptoms. Samples labeled as INT-B1, INT-B2 and INT-B3 are each a pool of sera from 10 vaccinated subjects. The color intensity designates the number of peptides of the indicated coronaviruses with a signal intensity threshold above 25'000

SARS-CoV-2 infected patients as well as vaccinated not-infected individuals developed cross-reactive antibodies against peptides of MERS-CoV, SARS-CoV and other coronaviruses (Fig. 2).

The immunodominant epitopes derived from the seven human coronaviruses, which are differentially recognized by the sera of diverse patient groups, are listed in the Additional file 2: Table S2. Overall, sera from patients with severe symptoms exhibited much more reactivity against SARS-CoV-2 and SARS-CoV in comparison with those from pediatric and mild/moderate symptoms patients (Fig. 2).

By setting the signal threshold above 25′000 no significant reactivity to SARS-CoV-2 peptides was detected in the vaccinated group compared to infected subjects, suggesting either that the first dose of vaccine induced low antibody levels (samples were collected 15–20 days after vaccination), or that vaccine-induced antibodies preferentially target conformational epitopes rather than linear epitopes.

Antibodies against N and S proteins of other coronaviruses (SARS-CoV, MERS, 229E, OC43, NL63 and HKU1) were detected in all groups without a significant correlation with the levels of SARS-CoV-2 fluorescent signals. However, the majority of samples reacted against peptides of MERS, a virus that has never been spread in Western countries, suggesting a cross-reaction of antibodies raised against different coronavirus proteins (Additional file 10: Fig. S5). In addition, all SARS-CoV-2 positive patients and vaccinated subjects exhibited high antibody response to S antigens of 229E, HKU1, NL63, OC43, and SARS-CoV. On the other end, a variable reactivity against N peptides of all coronaviruses was observed between patients in each group, but the signal intensities were generally low in vaccinated non-infected subjects.

The percentage of individuals who developed IgGs against N and S proteins of all coronaviruses is reported in Additional file 3: Table S3. Overall, the majority of subjects generated more anti-S than anti-N antibodies for all common coronaviruses. On the contrary, the patients with severe symptoms developed a stronger IgG response against linear peptides of SARS-CoV-2 N protein than S protein compared to other patient groups (Additional file 3: Table S3, Additional file 10: Figure S5). Moreover, children developed a stronger immunoreaction against other human coronaviruses than SARS-CoV-2.

### Identification of conserved immunodominant amino acid sequences between SARS-CoV-2 and human coronaviruses SARS-CoV, MERS-CoV, OC43, 229E, HKU1 and NL63

In order to determine whether SARS-CoV-2 antigens elicited cross-reactive antibodies against other coronaviruses, we performed homology search and amino acid sequence alignments of SARS-CoV-2 immunodominant peptides with other human coronaviruses (Table 3).

**Table 3:** Alignment of SARSCoV-2 peptides with the corresponding amino acid sequences of other human coronaviruses, which showed significant high signals with (A) adults and (B) children sera. Homologous sequences are marked in bold

A)				
SARS-CoV-2	NCAP	_157_I**VLQLPQGTTLPKG**F**YAEG**_175_	_221_**LLLLDRLNQLESK**MS_235_	_393_**TLLPAAD**L**DDFS**K**Q**L_407_
SARS-CoV	NCAP	_157_AT**VLQLPQGTTLPKGYAEG**_175_	_221_A**LLLLDRLNQLESK**V_235_	_393_V**TLLPAAD**M**DDFS**R**Q**_407_
SARS-CoV-2	SPIKE		_1145_LD**SFKEELDKYFKNH**_1159_	
SARS-CoV	SPIKE		_1129_**SFKEELDKYFKNH**TS_1143_	
MERS	SPIKE		_1225_NSTGID**F**QD**ELD**EF**F**_1243_	

B)				
SARS-CoV-2	NCAP	_109_**YFYYLG**TGPEAG**L**PY_123_		
SARS-CoV	NCAP	_101_KMKELSPRW**YFYYLG**_115_		
OC43	NCAP	_113_DGNQRQLLPRW**YFYY**_127_ _121_PRW**YFYYLGTGP**HAK_135_		
MERS	NCAP	_89_NGIKQLAPRW**YFYY**T_103_ _93_QLAPRW**YFYY**T**GTGP**_107_		
NL63	NCAP	_77_H**FYYLGTGP**HKD**L**KF_91_		
SARS-CoV-2	SPIKE	_553_**T**E**S**N**K**K**F**L**PFQQFGR**_567_	_1201_QE**LG**K**YE**Q**Y**I**KWPWY**_1215_	
SARS-CoV	SPIKE	_537_VL**T**P**S**S**K**R**F**Q**PFQQF**_551_		
OC43	SPIKE		_1285_KDI**G**T**YE**Y**Y**V**KWPWY**_1299_	
NL63	SPIKE		_1285_LLNRF**E**N**Y**I**KWPW**WV_1299_	
229E			_1105_LNRV**E**T**Y**I**KWPW**WVW_1119_	

**Fig. 3:**
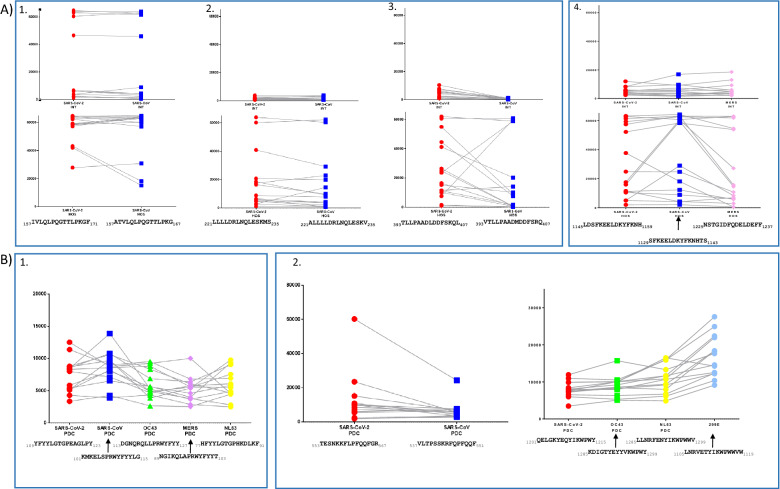
**A** SARS-CoV-2 N protein antibodies cross-react with SARS-CoV N protein (panels **A1**, **A2**, **A3**) and S protein (panel **A4**) in adults; **B** SARS-CoV-2 N protein antibodies cross-react with SARS-CoV N (panel **B1** and S **B2** proteins in children

Among these, six SARS-CoV-2 peptides showed high sequence similarity to the corresponding domains of SARS-CoV and one peptide to the OC43, NL63 and 229E epitopes. Three peptides, namely SARS-CoV-2 N aa 109–123, S aa 553–567 and S aa1201-1215, showed high reactivity with children sera, but not with adult specimens (Additional file 11: Figure S6-B). On the other hand, four peptides, namely SARS-CoV-2 N aa 157–175, N aa 221–235, N 393–407 and S aa 1145–1159, showed high signals only when probed with adult sera. Overall, comparison of fluorescent signals, obtained by probing each sample against SARS-CoV-2 immunodominant peptides and corresponding coronavirus epitopes, showed similar values indicating that anti-SARS-Cov-2 antibodies were cross-reactive with other human coronaviruses epitopes (Fig. 3A (1-4) and B(1-3))(Additional file 11: Figure S6-A). Only the 229E S aa 1105–1119 peptide probed with the children sera was more reactive than SARS-CoV-2 S aa 1201–1215, probably due to a recent infection with 229E strain (Additional file 11: Figure S6-B). The antibody response was always significantly higher in severe symptoms patients (Fig. 3 A-2, A-3, A-4).

### Clinical laboratory findings in COVID-19 patients

Hematological and inflammatory parameters of children and hospitalized patients have been reported in Additional files 4, 5: Table S4 and S5. The majority of patients with severe symptoms who were admitted to intensive care experienced hyperglycemia (100%), increased prothrombin activity (100%) as well as high levels of D-Dimer (69%) and urea nitrogen (80%). These findings are in agreement with previous studies showing that severe COVID-19 has been reported to be associated with increased blood glucose and its monitoring improves prognosis [27, 28].

Among children presenting with SARS-CoV-2 related symptoms, 60% had high D-Dimer values, 80% had high PCR levels and 100% had high levels of PCT (Additional file 5: Table S5).

## Discussion

Antibody levels elicited by SARS-CoV-2 proteins following natural infection or vaccine administration are so far considered the strongest correlates of protection from virus reinfection and COVID-19 disease severity [29, 30]. In addition, COVID-19-related hospitalization or death is strongly reduced by the administration of anti-SARS-CoV-2 monoclonal antibodies targeting the S protein [31–34]. However, while some studies showed that an abundance of immunoglobulins against SARS-CoV-2 was associated with reduced disease severity and faster recovery, other findings showed that high antibody titers were associated with severe COVID-19 symptoms [35–38].

We have analyzed the antibody response in three cohorts of SARS-CoV-2 infected subjects including children, adults with mild/moderate symptoms and hospitalized patients with severe COVID-19. The analysis of serum immunoreactivity to 5,892 linear peptides, corresponding to the whole SARS-CoV-2 proteome as well as to the N and S proteins of six human coronaviruses, showed that all SARS-CoV-2 infected subjects exhibit a robust IgG response mainly against SARS-CoV-2 N and S peptides. These results are in agreement with previous studies reporting N and S as the main antigenic proteins in SARS-CoV-2 infected individuals [39–45]. In addition, patients with severe symptoms developed the highest antibody levels against SARS-CoV-2 N epitopes, while children have the lowest, suggesting an age-dependent antibody production against the N protein. The reduced magnitude of N antibodies in children has been prior reported in other studies and suggested to be related to a lower virus replication and limited release of N proteins in children [46, 47]. Several studies have shown that SARS-CoV-2 receptor ACE2 has a lower expression in the lungs of children compared to adults and that low ACE2 levels could be related to reduced virus replication and decreased susceptibility of children to Covid-19 [48].

In accordance with previous studies, we observed that children developed higher levels of antibodies against S protein compared to adults [40]. One explanation for different IgG responses against SARS-CoV-2 proteins between children and adults could be the higher rate of infection with other human coronaviruses and related stronger cross-immunity in children [49]. Accordingly, we observed that in children, but not in adults, the IgG response against the coronavirus 229E S aa 1105–1119 epitope was stronger than the homologous SARS-CoV-2 S aa 1201–1215 domain suggesting a pre-existing cross-protective immunity against SARS-CoV-2. In addition, several studies evaluating the cytokine profile in SARS-CoV-2 infected subjects showed that children in comparison with adults have lower cytokine levels, particularly IL-8, IL-6, and MCP-1, and those antibodies against specific SARS-CoV-2 proteins, such as ORF8, inversely correlate with IL-6 levels suggesting a negative feedback loop between anti-viral antibody production and expression of inflammatory factors [50]. Moreover, Loske et al., by performing single-cell analysis of the upper airway observed in the epithelial cells, macrophages and dendritic cells a higher expression of MDA5 and RIG-I pattern recognition receptors in SARS-CoV-2-negative and SARS-CoV-2-positive children compared to adults [51]. In addition, distinct immune cell types, including KLRC1 (NKG2A)^+^ cytotoxic T cells and a CD8^+^ T cell population, were identified in the airway immune cells of children possibly associated with stronger early innate response to SARS-CoV-2 infection [51].

Several immunodominant domains in SARS-CoV-2 N and S proteins have been previously identified by means of different methodologies [39, 52, 53]. In our study, by using a peptide microarray analysis, we identified eight linear peptides of SARS-CoV-2 N and S proteins, which have been found to elicit higher antibody levels in severe symptoms compared to mild/moderate symptoms adult patients. Among these, two overlapping sequences (aa 157–171 and aa 161–175) of the N protein have been previously shown to be associated with severe disease also in other studies [53, 54]. Indeed, Amrun et al. reported a direct relationship between disease severity and antibody abundance against the two above described epitopes by analyzing pooled sera of COVID-19 patients [54]. Accordingly, Gregory et al. reported that the antibody levels against the N domain aa 161–171 were specifically increased in patients with SARS-CoV-2-related severe disease [53]. Furthermore, an additional epitope (aa 221–235) of the N protein has shown to be highly reactive when probed with the sera of patients with severe disease in our and in other published studies [55].

Our findings showed that sera from hospitalized patients were also strongly reactive against three peptides in the S1 subunit, one located in the receptor binding domain (aa 557–571) and two in the fusion domain (aa 785–799 and aa 789–803), as well as one peptide (aa 1145–1159) in the S2 subunit. Previous studies only identified the aa 785–799 peptide as one of the immunodominant epitopes of S protein in COVID-19 patients [19, 26, 55].

The VirScan platform, based on the immunoprecipitation and DNA sequencing of a phage display library spanning the SARS-CoV-2 and all other human coronaviruses proteomes, has been used to profile the antibody response to SARS-CoV-2 in 19 patients and seven unexposed control subjects [42]. Among the reactive epitopes of SARS-CoV-2 N and S proteins, which were specifically recognized by several SARS-CoV-2 infected subjects, there were sequences comprising all the N epitopes (aa 157–171, aa 161–175, aa 221–235 and aa 393–407) and the S epitopes (aa 557–571, aa 785–799, aa 789–803 and aa 1145–159) identified in our study [42].

In addition, we have identified a previous unreported R1A-R1AB epitope (aa 249–263), which elicited IgG response in all SARS-CoV-2 infected patients, but the antibody abundance was higher in the asymptomatic compared to mild/moderate symptoms patients. Of note, this peptide has high homology to the amino acid sequences EIKKAKKF and SPFEIKLA, contained in the diphtheria, tetanus, and pertussis vaccines (DTP), which have been suggested to cause a cross-reactive immunity to SARS-CoV-2 [56]. However, given the limited number of asymptomatic subject in our study and their antibody levels against the DTP vaccine we cannot conclude that a cross-reactivity response between SARS-CoV-2 and DTP has protected these subjects from SARS-CoV-2 symptoms.

Previous studies evaluating the humoral immune response elicited by SARS-CoV-2 infection in children and adults showed several differences in the viral proteins targeted by the antibodies [57]. Hachim et al. reported that SARS-CoV-2 infected children produced lower levels of antibodies targeting S, M, ORF3a, ORF7a, ORF7b, comparable levels to ORF8 and elevated antibody titers to E protein than adults [57]. On the other hand, we identified 30 distinct peptides in the S, N, M, R1A-R1AB and accessory proteins NS7A and NS8, which elicited significant higher antibody levels in children less than 8 years old compared with adults diagnosed with mild/moderate symptoms. It has been suggested that children may be protected from SARS-CoV-2 through cross-reactive immunity elicited by vaccinations. However, among these peptides only the M epitope (aa 5–19) has been shown to share homology with the VEELKKLL sequence contained in the DTP vaccines and suggested to elicit cross-reactive adaptive immunity to SARS-CoV-2 [56].

In silico studies predicting SARS-CoV-2 immunogenicity suggested the existence of cross-reactive B-cell and T-cell domains between SARS-CoV-2 and common human coronaviruses [26]. Importantly, the preexisting immune response elicited by common cold coronaviruses to SARS-CoV-2 has been proposed to influence the clinical course of COVID-19. Several T-cell epitopes and B-cell cross-reactivity have been identified [58, 59]. However, common cold coronaviruses are known to induce transient humoral immune response with antibodies without neutralizing activity against SARS-CoV-2 [60, 61].

We found that all sera from SARS-CoV-2 infected and vaccinated adult subjects reacted against S and N protein of 229E, HKU1, NL63, OC43, and SARS-CoV. However, the fluorescent signals were lower compared to the SARS-CoV-2 IgG response. On the contrary, children showed a stronger antibody response against N and S proteins of other coronaviruses compared to SARS-CoV-2. The higher reactivity to MERS, a virus that has never spread to Western countries, suggests the occurrence of non-specific binding to MERS proteins by children and adults IgGs.

Among the immunodominant epitopes of SARS-CoV-2, which had a significant binding difference between mild/moderate and severe symptoms adult patients, three peptides of the N protein (aa 157–175, 221–235 and 393–407) and one of S protein (aa 1145–1159) showed a relevant homology with the corresponding domain in SARS-CoV. Antibody levels towards SARS-CoV-2 and homologous SARS-CoV domains were correspondingly high in severe symptoms patients and low in mild/moderate symptoms patients. Accordingly, two immunodominant peptides of SARS-CoV-2 N (aa 109–123, aa 221–235) and one of S (553–567) showed similar results with children sera. On the other hand, sera from children were found to bind significantly more the S peptide aa 1105–1119 of the 229E coronavirus than corresponding antigens of SARS-CoV-2 and other coronaviruses. Other studies reported that infection with SARS-CoV-2 elicits or boosts the level of antibodies that bind to the N and S proteins of other coronaviruses, including SARS-CoV, MERS-CoV, HCoV-NL63, and HCoV-OC43, particularly in patients with severe symptoms [42, 62, 63]. However, our results showed that the cross-reactive antibody responses against several epitopes shared between SARS-CoV-2 and other coronaviruses largely depends on the abundance of antibodies elicited by SARS-CoV-2. Conversely, the higher level of antibodies raised against 229E epitopes compared to SARS-CoV-2 in children may be associated with a protective cross-reactive immune response. However, a prior study evaluating the infection of seasonal coronaviruses in SARS-CoV-2 infected children with or without multisystem inflammatory syndrome have not observed any interference with SARS-CoV-2 clinical course [64].

Our study has some limitations such as 1) the retrospective design that does not allow proper analysis of several laboratory parameters such as the inflammatory markers; 2) the range of time from diagnosis to sera collection varying up to 20 days in the mild/moderate symptoms group; 3) the limited number of patients in some groups may have underestimated peptides with statistically significant antibody reactivity, and 4) only antibodies targeting linear peptides, instead of the natively folded antigens, have been measured by peptide-microarray analysis.

## Conclusion

In conclusion, the study of the antibody levels to peptides covering the whole SARS-CoV-2 proteome allowed identifying specific immunodominant amino acid sequences in children and adult patients. In addition, we have confirmed previous studies showing that the antibody abundance towards specific sequences of N and S proteins are associated with severe disease in adult patients. Overall, the peptides associated with immune response in children, which in most cases do not exhibit severe symptoms, may be valuable targets for SARS-CoV-2 vaccines or diagnostics and provide insights into the viral pathogenesis.

## Supplementary Information

**Additional file 1: Table S1.** Proteins represented on RepliTopeTM Antigen Collection Pan-Coronavirus microarray.

**Additional file 2: Table S2.** Supplementary. Immunodominant epitopes for adult groups (comparison between groups pediatric/mild/severe). Amino acids marked in red indicate overlapped sequences.

**Additional file 3: Table S3. **Percentage of individuals who developed IgG response against N and S proteins of the seven human coronaviruses in each patient group.

**Additional file 4: Table S4.** Clinical features of hospitalized adult patients with SARS-CoV-2 infection.

**Additional file 5: Table S5.** Clinical and laboratory parameters of children hospitalized with SARS-CoV-2 infection.

**Additional file 6: Fig. S1**. Heatmaps obtained by probing the arrays with serum samples towards SARS-CoV-2 S (a), N (b), M (h), ORF3a (f), NS7 (g) and N proteins of CVHSA (SARS-CoV, c), CVEMC(MERS, d) and 229E (e). Each column represents the reaction of a serum sample against all viral epitopes. The rows represent overlapping peptides from the respective protein. Color codes indicate strong signals with red squares, low signals with yellow squares and no signal with white squares. Sample groups: 1) negative control obtained by probing the arrays with secondary antibody only, 2) Children samples (PDC), 3) adults samples with mild/moderate symptoms (INT-A) and vaccinated adults samples (VAX-B), 4) adults samples with severe symptoms (HOS).

**Additional file 7: Fig.S2.** A) Percentage of patients in each group, which reacted against peptides of S protein. B) Percentage of patients in each group, which reacted against peptides of M protein.

**Additional file 8: Fig. S3. **Serum samples reactivity against SARS-CoV-2 epitopes: Samples from adults (A) and from children (B) with high reactivity towards epitopes of different proteins of SARS-CoV-2.

**Additional file 9: Fig.S4**: A) Percentage of patients between groups who developed antibodies against epitopes of N protein, B) percentage of patients between groups who developed antibodies against epitopes of S protein; C) percentage of patients between group who developed antibodies against epitopes of M protein; D) Number of patients who developed antibodies against the most reactive epitopes. All HOS and 5 INT patients with moderate symptoms developed antibodies against two peptides from N protein (aa 157-171 and aa 161-175), all HOS and PDC developed antibodies against two peptides of S protein (aa 553-567 and aa 557-571) and against one peptide of M protein(aa 5-19); all HOS developed antibodies against the peptide aa 785-799 of S protein.

**Additional file 10: Fig. S5. **Barplot showing the fraction of serum signals reacting towards N and S proteins of MERS and common-cold coronaviruses obtained by setting the threshold above 10,000. Control samples are reaction controls with the secondary antibody only (n=3), the PDC identifies samples from SARS-CoV-2 positive children, INT identifies samples from adults with mild/moderate symptoms and HOS the hospitalized patients with severe symptoms. Samples labeled as VAX-B1, VAX-B2 and VAX-B3 are each a pool of sera from 10 vaccinated subjects.

**Additional file 11: Fig. S6. **Antibodies reactivity against MERS and common-cold coronaviruses epitopes: adult serum samples (A) and children serum samples (B) with high reactivity against epitopes of different coronaviruses.

## Data Availability

The raw data are available through Zenodo at 7486780.

## References

[1] V'Kovski P, Kratzel A, Steiner S, Stalder H, Thiel V (2021). Coronavirus biology and replication: implications for SARS-CoV-2. Nat Rev Microbiol.

[2] Pal M, Berhanu G, Desalegn C, Kandi V (2020). Severe acute respiratory syndrome coronavirus-2 (SARS-CoV-2): an update. Cureus.

[3] Alsobaie S (2021). Understanding the molecular biology of SARS-CoV-2 and the COVID-19 pandemic: a review. Infect Drug Resist.

[4] Buonaguro FM, Ascierto PA, Morse GD, Buonaguro L, Puzanov I, Tornesello ML, Brechot C, Gallo RC (2020). Covid-19: time for a paradigm change. Rev Med Virol.

[5] Poudel K, Subedi P (2020). Impact of COVID-19 pandemic on socioeconomic and mental health aspects in Nepal. Int J Soc Psychiatry.

[6] Fung TS, Liu DX (2019). Human coronavirus: host-pathogen interaction. Annu Rev Microbiol.

[7] Yang H, Rao Z (2021). Structural biology of SARS-CoV-2 and implications for therapeutic development. Nat Rev Microbiol.

[8] Wu CR, Yin WC, Jiang Y, Xu HE (2022). Structure genomics of SARS-CoV-2 and its Omicron variant: drug design templates for COVID-19. Acta Pharmacol Sin.

[9] Gordon DE, Jang GM, Bouhaddou M, Xu J, Obernier K, White KM, O'Meara MJ, Rezelj VV, Guo JZ, Swaney DL (2020). A SARS-CoV-2 protein interaction map reveals targets for drug repurposing. Nature.

[10] Redondo N, Zaldivar-Lopez S, Garrido JJ, Montoya M (2021). SARS-CoV-2 accessory proteins in viral pathogenesis: knowns and unknowns. Front Immunol.

[11] Meyer B, Drosten C, Muller MA (2014). Serological assays for emerging coronaviruses: challenges and pitfalls. Virus Res.

[12] Jackson CB, Farzan M, Chen B, Choe H (2022). Mechanisms of SARS-CoV-2 entry into cells. Nat Rev Mol Cell Biol.

[13] Ord M, Faustova I, Loog M (2020). The sequence at Spike S1/S2 site enables cleavage by furin and phospho-regulation in SARS-CoV2 but not in SARS-CoV1 or MERS-CoV. Sci Rep.

[14] Salamanna F, Maglio M, Landini MP, Fini M (2020). Body localization of ACE-2: on the trail of the keyhole of SARS-CoV-2. Front Med (Lausanne).

[15] Lan J, Ge J, Yu J, Shan S, Zhou H, Fan S, Zhang Q, Shi X, Wang Q, Zhang L (2020). Structure of the SARS-CoV-2 spike receptor-binding domain bound to the ACE2 receptor. Nature.

[16] Premkumar L, Segovia-Chumbez B, Jadi R, Martinez DR, Raut R, Markmann A, Cornaby C, Bartelt L, Weiss S, Park Y (2020). The receptor binding domain of the viral spike protein is an immunodominant and highly specific target of antibodies in SARS-CoV-2 patients. Sci Immunol.

[17] Piccoli L, Park YJ, Tortorici MA, Czudnochowski N, Walls AC, Beltramello M, Silacci-Fregni C, Pinto D, Rosen LE, Bowen JE (2020). Mapping neutralizing and immunodominant sites on the SARS-CoV-2 spike receptor-binding domain by structure-guided high-resolution serology. Cell.

[18] Astuti I, Ysrafil (2020). Severe acute respiratory syndrome coronavirus 2 (SARS-CoV-2): an overview of viral structure and host response. Diabetes Metab Syndr.

[19] Li Y, Ma ML, Lei Q, Wang F, Hong W, Lai DY, Hou H, Xu ZW, Zhang B, Chen H (2021). Linear epitope landscape of the SARS-CoV-2 Spike protein constructed from 1,051 COVID-19 patients. Cell Rep.

[20] Caddy SL, Vaysburd M, Papa G, Wing M, O'Connell K, Stoycheva D, Foss S, Terje Andersen J, Oxenius A, James LC (2021). Viral nucleoprotein antibodies activate TRIM21 and induce T cell immunity. EMBO J.

[21] Girona-Alarcon M, Bobillo-Perez S, Sole-Ribalta A, Hernandez L, Guitart C, Suarez R, Balaguer M, Cambra FJ, Jordan I, Group KI-Cs (2021). The different manifestations of COVID-19 in adults and children: a cohort study in an intensive care unit. BMC Infect Dis.

[22] Li J, Thoon KC, Chong CY, Maiwald M, Kam KQ, Nadua K, Tan NW, Yung CF (2020). Comparative analysis of symptomatic and asymptomatic SARS-CoV-2 infection in children. Ann Acad Med Singap.

[23] Petrara MR, Bonfante F, Costenaro P, Cantarutti A, Carmona F, Ruffoni E, Di Chiara C, Zanchetta M, Barzon L, Dona D (2021). Asymptomatic and mild SARS-CoV-2 infections elicit lower immune activation and higher specific neutralizing antibodies in children than in adults. Front Immunol.

[24] Stephenson KE, Neubauer GH, Reimer U, Pawlowski N, Knaute T, Zerweck J, Korber BT, Barouch DH (2015). Quantification of the epitope diversity of HIV-1-specific binding antibodies by peptide microarrays for global HIV-1 vaccine development. J Immunol Methods.

[25] Frank R (2002). The SPOT-synthesis technique. Synthetic peptide arrays on membrane supports–principles and applications. J Immunol Methods.

[26] Holenya P, Lange PJ, Reimer U, Woltersdorf W, Panterodt T, Glas M, Wasner M, Eckey M, Drosch M, Hollidt JM (2021). Peptide microarray-based analysis of antibody responses to SARS-CoV-2 identifies unique epitopes with potential for diagnostic test development. Eur J Immunol.

[27] Chen J, Wu C, Wang X, Yu J, Sun Z (2020). The impact of COVID-19 on blood glucose: a systematic review and meta-analysis. Front Endocrinol (Lausanne).

[28] Xiao F, Zhou YC, Zhang MB, Chen D, Peng SL, Tang HN, Li L, Tang CY, Liu JY, Li B (2022). Hyperglycemia and blood glucose deterioration are risk factors for severe COVID-19 with diabetes: a two-center cohort study. J Med Virol.

[29] Khoury DS, Cromer D, Reynaldi A, Schlub TE, Wheatley AK, Juno JA, Subbarao K, Kent SJ, Triccas JA, Davenport MP (2021). Neutralizing antibody levels are highly predictive of immune protection from symptomatic SARS-CoV-2 infection. Nat Med.

[30] Hall VJ, Foulkes S, Charlett A, Atti A, Monk EJM, Simmons R, Wellington E, Cole MJ, Saei A, Oguti B (2021). SARS-CoV-2 infection rates of antibody-positive compared with antibody-negative health-care workers in England: a large, multicentre, prospective cohort study (SIREN). Lancet.

[31] Wheatley AK, Pymm P, Esterbauer R, Dietrich MH, Lee WS, Drew D, Kelly HG, Chan LJ, Mordant FL, Black KA (2021). Landscape of human antibody recognition of the SARS-CoV-2 receptor binding domain. Cell Rep.

[32] Hirsch C, Park YS, Piechotta V, Chai KL, Estcourt LJ, Monsef I, Salomon S, Wood EM, So-Osman C, McQuilten Z (2022). SARS-CoV-2-neutralising monoclonal antibodies to prevent COVID-19. Cochrane Database Syst Rev.

[33] Bloch EM, Shoham S, Casadevall A, Sachais BS, Shaz B, Winters JL, van Buskirk C, Grossman BJ, Joyner M, Henderson JP (2020). Deployment of convalescent plasma for the prevention and treatment of COVID-19. J Clin Invest.

[34] Kudlay D, Svistunov A (2022). COVID-19 vaccines: an overview of different platforms. Bioengineering (Basel).

[35] Garcia-Beltran WF, Lam EC, Astudillo MG, Yang D, Miller TE, Feldman J, Hauser BM, Caradonna TM, Clayton KL, Nitido AD (2021). COVID-19-neutralizing antibodies predict disease severity and survival. Cell.

[36] Atyeo C, Fischinger S, Zohar T, Slein MD, Burke J, Loos C, McCulloch DJ, Newman KL, Wolf C, Yu J (2020). Distinct early serological signatures track with SARS-CoV-2 survival. Immunity.

[37] Ren L, Zhang L, Chang D, Wang J, Hu Y, Chen H, Guo L, Wu C, Wang C, Wang Y (2020). The kinetics of humoral response and its relationship with the disease severity in COVID-19. Commun Biol.

[38] Guthmiller JJ, Stovicek O, Wang J, Changrob S, Li L, Halfmann P, Zheng NY, Utset H, Stamper CT, Dugan HL (2021). SARS-CoV-2 infection severity is linked to superior humoral immunity against the Spike. MBio.

[39] Voss C, Esmail S, Liu X, Knauer MJ, Ackloo S, Kaneko T, Lowes L, Stogios P, Seitova A, Hutchinson A (2021). Epitope-specific antibody responses differentiate COVID-19 outcomes and variants of concern. JCI Insight.

[40] Weisberg SP, Connors TJ, Zhu Y, Baldwin MR, Lin WH, Wontakal S, Szabo PA, Wells SB, Dogra P, Gray J (2021). Distinct antibody responses to SARS-CoV-2 in children and adults across the COVID-19 clinical spectrum. Nat Immunol.

[41] Lucchese G (2020). Epitopes for a 2019-nCoV vaccine. Cell Mol Immunol.

[42] Shrock E, Fujimura E, Kula T, Timms RT, Lee IH, Leng Y, Robinson ML, Sie BM, Li MZ, Chen Y (2020). Viral epitope profiling of COVID-19 patients reveals cross-reactivity and correlates of severity. Science.

[43] Long QX, Liu BZ, Deng HJ, Wu GC, Deng K, Chen YK, Liao P, Qiu JF, Lin Y, Cai XF (2020). Antibody responses to SARS-CoV-2 in patients with COVID-19. Nat Med.

[44] Manenti A, Gianchecchi E, Dapporto F, Leonardi M, Cantaloni P, Fattorini F, Piu P, Bollati V, Pastorino U, Apolone G (2022). Evaluation and correlation between SARS-CoV-2 neutralizing and binding antibodies in convalescent and vaccinated subjects. J Immunol Methods.

[45] Isgro MA, Trillo G, Russo L, Tornesello AL, Buonaguro L, Tornesello ML, Miscio L, Normanno N, Bianchi AAM, Buonaguro FM (2022). Bimodal antibody-titer decline following BNT162b2 mRNA anti-SARS-CoV-2 vaccination in healthcare workers of the INT - IRCCS "Fondazione Pascale" Cancer Center (Naples, Italy). Infect Agent Cancer.

[46] Allen N, Brady M, Carrion Martin AI, Domegan L, Walsh C, Doherty L, Riain UN, Bergin C, Fleming C, Conlon N (2021). Serological markers of SARS-CoV-2 infection; anti-nucleocapsid antibody positivity may not be the ideal marker of natural infection in vaccinated individuals. J Infect.

[47] Hachim A, Gu H, Kavian O, Mori M, Kwan MYW, Chan WH, Yau YS, Chiu SS, Tsang OTY, Hui DSC (2022). SARS-CoV-2 accessory proteins reveal distinct serological signatures in children. Nat Commun.

[48] Silva MG, Falcoff NL, Corradi GR, Di Camillo N, Seguel RF, Tabaj GC, Guman GR, de Matteo E, Nunez M, Gironacci MM (2022). Effect of age on human ACE2 and ACE2-expressing alveolar type II cells levels. Pediatr Res.

[49] Monto AS, DeJonge PM, Callear AP, Bazzi LA, Capriola SB, Malosh RE, Martin ET, Petrie JG (2020). Coronavirus occurrence and transmission over 8 years in the HIVE Cohort of households in Michigan. J Infect Dis.

[50] Chou J, Thomas PG, Randolph AG (2022). Immunology of SARS-CoV-2 infection in children. Nat Immunol.

[51] Loske J, Rohmel J, Lukassen S, Stricker S, Magalhaes VG, Liebig J, Chua RL, Thurmann L, Messingschlager M, Seegebarth A (2022). Pre-activated antiviral innate immunity in the upper airways controls early SARS-CoV-2 infection in children. Nat Biotechnol.

[52] Li Y, Lai DY, Zhang HN, Jiang HW, Tian X, Ma ML, Qi H, Meng QF, Guo SJ, Wu Y (2020). Linear epitopes of SARS-CoV-2 spike protein elicit neutralizing antibodies in COVID-19 patients. Cell Mol Immunol.

[53] Gregory DJ, Vannier A, Duey AH, Roady TJ, Dzeng RK, Pavlovic MN, Chapin MH, Mukherjee S, Wilmot H, Chronos N (2022). Repertoires of SARS-CoV-2 epitopes targeted by antibodies vary according to severity of COVID-19. Virulence.

[54] Amrun SN, Lee CY, Lee B, Fong SW, Young BE, Chee RS, Yeo NK, Torres-Ruesta A, Carissimo G, Poh CM (2020). Linear B-cell epitopes in the spike and nucleocapsid proteins as markers of SARS-CoV-2 exposure and disease severity. EBioMedicine.

[55] Mishra N, Huang X, Joshi S, Guo C, Ng J, Thakkar R, Wu Y, Dong X, Li Q, Pinapati RS (2021). Immunoreactive peptide maps of SARS-CoV-2. Commun Biol.

[56] Reche PA (2020). Potential cross-reactive immunity to SARS-CoV-2 from common human pathogens and vaccines. Front Immunol.

[57] Ravichandran S, Tang J, Grubbs G, Lee Y, Pourhashemi S, Hussaini L, Lapp SA, Jerris RC, Singh V, Chahroudi A (2021). SARS-CoV-2 immune repertoire in MIS-C and pediatric COVID-19. Nat Immunol.

[58] Ng KW, Faulkner N, Cornish GH, Rosa A, Harvey R, Hussain S, Ulferts R, Earl C, Wrobel AG, Benton DJ (2020). Preexisting and de novo humoral immunity to SARS-CoV-2 in humans. Science.

[59] Bonifacius A, Tischer-Zimmermann S, Dragon AC, Gussarow D, Vogel A, Krettek U, Godecke N, Yilmaz M, Kraft ARM, Hoeper MM (2021). COVID-19 immune signatures reveal stable antiviral T cell function despite declining humoral responses. Immunity.

[60] Huang AT, Garcia-Carreras B, Hitchings MDT, Yang B, Katzelnick LC, Rattigan SM, Borgert BA, Moreno CA, Solomon BD, Trimmer-Smith L (2020). A systematic review of antibody mediated immunity to coronaviruses: kinetics, correlates of protection, and association with severity. Nat Commun.

[61] Lv H, Wu NC, Tsang OT, Yuan M, Perera R, Leung WS, So RTY, Chan JMC, Yip GK, Chik TSH (2020). Cross-reactive antibody response between SARS-CoV-2 and SARS-CoV Infections. Cell Rep.

[62] Ladner JT, Henson SN, Boyle AS, Engelbrektson AL, Fink ZW, Rahee F, D'Ambrozio J, Schaecher KE, Stone M, Dong W (2021). Epitope-resolved profiling of the SARS-CoV-2 antibody response identifies cross-reactivity with endemic human coronaviruses. Cell Rep Med.

[63] Camerini D, Randall AZ, Trappl-Kimmons K, Oberai A, Hung C, Edgar J, Shandling A, Huynh V, Teng AA, Hermanson G (2021). Mapping SARS-CoV-2 antibody epitopes in COVID-19 patients with a multi-coronavirus protein microarray. Microbiol Spectr.

[64] Sermet-Gaudelus I, Temmam S, Huon C, Behillil S, Gajdos V, Bigot T, Lurier T, Chretien D, Backovic M, Delaunay-Moisan A (2021). Prior infection by seasonal coronaviruses, as assessed by serology, does not prevent SARS-CoV-2 infection and disease in children, France, April to June 2020. Euro Surveill.

