# Supplementary material for: Immune profiling of SARS-CoV-2 epitopes in asymptomatic and symptomatic pediatric and adult patients
Source: J Transl Med. 2023 Feb 14;21:123. doi: 10.1186/s12967-023-03963-5 (PMC9927035; doi:10.1186/s12967-023-03963-5)
Supplement: Supplementary file 1 — Additional file 1: Table S1. Proteins represented on RepliTopeTM Antigen Collection Pan-Coronavirus microarray. [file 12967_2023_3963_MOESM1_ESM.pdf]

Table 1S: Proteins represented on RepliTope™ Antigen Collection Pan-Coronavirus microarray

**ID**	**Peptides**	**Protein**	**organism**
Spike_SARS2	316	Spike glycoprotein	SARS-CoV-2
NCAP_SARS2	102	Nucleoprotein	SARS-CoV-2
VEMP_SARS2 (E protein)	16	Envelope small membrane protein	SARS-CoV-2
VME1_SARS2	53	Membrane protein	SARS-CoV-2
*R1AB_SARS2	1772	Replicase polyprotein 1ab	SARS-CoV-2
*R1A_SARS2	1099[1)]	Replicase polyprotein 1a	SARS-CoV-2
AP3A_SARS2 (ORF3a)	66	Protein 3a	SARS-CoV-2
NS6_SARS2 (ORF6)	13	Non-structural protein 6	SARS-CoV-2
NS7A_SARS2 (ORF7a)	28	Protein 7a	SARS-CoV-2
NS7B_SARS2 (ORF7B)	8	Non-structural protein 7b	SARS-CoV-2
NS8_SARS2 (ORF8B)	28	Non-structural protein 8	SARS-CoV-2
ORF9B_SARS2 (ORF9B)	22	Protein 9b	SARS-CoV-2
Y14_SARS2 (ORF9C)	16	Uncharacterized protein 14	SARS-CoV-2
A0A663DJA2_SARS2	7	Orf 10 protein	SARS-CoV-2
NCAP_CVEMC	100	Nucleoprotein	MERS
SPIKE_CVEMC	336	Spike glycoprotein	MERS
VEMP_CVEMC	18	Envelope small membrane protein	MERS
VME1_CVEMC	52	Membrane protein	MERS
NCAP_CVHSA	103	Nucleoprotein	SARS-CoV
SPIKE_CVHSA	311	Spike glycoprotein	SARS-CoV
VEMP_CVHSA	17	Envelope small membrane protein	SARS-CoV
VME1_CVHSA	53	Membrane protein	SARS-CoV
NCAP_CVH22	95	Nucleoprotein	229E
SPIKE_CVH22	291	Spike glycoprotein	229E
VEMP_CVH22	17	Envelope small membrane protein	229E
VME1_CVH22	54	Membrane protein	229E
NCAP_CVHNL	92	Nucleoprotein	NL63
SPIKE_CVHNL	337	Spike glycoprotein	NL63
VEMP_CVHNL	17	Envelope small membrane protein	NL63
VME1_CVHNL	54	Membrane protein	NL63
NCAP_CVHOC	110	Nucleoprotein	OC43
SPIKE_CVHOC	336	Spike glycoprotein	OC43
VEMP_CVHOC	19	Envelope small membrane protein	OC43
VME1_CVHOC	55	Membrane protein	OC43
NCAP_CVHN1	105[2)]	Nucleoprotein	UKU1(N1)
SPIKE_CVHN1	329[3)]	Spike glycoprotein	UKU1(N1)
VEMP_CVHN1	18[4)]	Envelope small membrane protein	UKU1(N1)
VME1_CVHN1	51[5)]	Membrane protein	UKU1(N1)
NCAP_CVHN2	105[2)]	Nucleoprotein	UKU1(N2)
SPIKE_CVHN2	327[3)]	Spike glycoprotein	UKU1(N2)
VEMP_CVHN2	18[4)]	Envelope small membrane protein	UKU1(N2)
VME1_CVHN2	51[5)]	Membrane protein	UKU1(N2)
NCAP_CVHN5	105[2)]	Nucleoprotein	UKU1(N5)
SPIKE_CVHN5	327[3)]	Spike glycoprotein	UKU1(N5)
VEMP_CVHN5	18[4)]	Envelope small membrane protein	UKU1(N5)
VME1_CVHN5	51[5)]	Membrane protein	UKU1(N5)

*include Nsp9, Nsp10, Nsp11, Nsp12, Nsp13, Nsp14, Nsp15, Nsporf16
