# Supplementary material for: Immune profiling of SARS-CoV-2 epitopes in asymptomatic and symptomatic pediatric and adult patients
Source: J Transl Med. 2023 Feb 14;21:123. doi: 10.1186/s12967-023-03963-5 (PMC9927035; doi:10.1186/s12967-023-03963-5)
Supplement: Supplementary file 2 — Additional file 2: Table S2. Supplementary. Immunodominant epitopes for adult groups (comparison between groups pediatric/mild/severe). Amino acids marked in red indicate overlapped sequences. [file 12967_2023_3963_MOESM2_ESM.pdf]

Table 2S Supplementary. Immunodominant epitopes for adult groups (comparison between groups pediatric/mild/severe). Amino acids marked in red indicate overlapped sequences.

Peptide	Organism	Protein	Sequence	p-value	Comparison groups	AUC
NCAP_SARS2_0157-0171	SARS-CoV-2	N	IVLQLPQGTTLPKGF	$9.9X10^{-3}$	Mild/Sev	0.773
NCAP_SARS2_0161-0175	SARS-CoV-2	N	LPQGTTLPKGFYAEG	$4.9X10^{-3}$	Mild/Sev	0.796
NCAP_SARS2_0221-0235	SARS-CoV-2	**N**	LLLLDRLNQLESKMS	**$1.6X10^{-5}$**	**Mild/Sev**	**0.924**
NCAP_SARS2_0393-0407	SARS-CoV-2	**N**	TLLPAADLDDFSKQL	**$1X10^{-3}$**	**Mild/Sev**	**0.84**
SPIKE_SARS2_0557-0571	SARS-CoV-2	**S**	KKFLPFQQFGRDIAD	**$8.6X10^{-3}$**	**MILD/SEV**	**0.778**
SPIKE_SARS2_0785-0799	SARS-CoV-2	**S**	VKQIYKTPPIKDFGG	**$1X10^{-4}$**	**Mild/Sev**	**0.889**
SPIKE_SARS2_0789-0803	SARS-CoV-2	S	YKTPPIKDFGGFNFS	$2X10^{-3}$	Mild/Sev	0.822
SPIKE_SARS2_1145-1159	SARS-CoV-2	**S**	LDSFKEELDKYFKNH	**$1.2X10^{-3}$**	**Mild/Sev**	**0.836**
R1A_R1AB_SARS2_0249-0263	SARS-CoV-2	NsP	YELQTPFEIKLAKKF	$4.8X10^{-3}$	Asym/mild	0.926
R1AB_SARS2_6073-6087	SARS-CoV-2	**NsP**	HLIPLMYKGLPWNVV	**$5.7X10^{-3}$**	**Mild/Sev**	**0.791**
NCAP_CVHSA_0157-0171	SARS-CoV	**N**	ATVLQLPQGTTLPKG	**$2.3X10^{-3}$**	**Mild/Sev**	**0.818**
NCAP_CVHSA_0161-0175	SARS-CoV	N	QLPQGTTLPKGFYAE	$6.6X10^{-3}$	Mild/Sev	0.787
NCAP_CVHSA_0221-0235	SARS-CoV	**N**	ALLLLDRLNQLESKV	**$6X10^{-4}$**	**Mild/Sev**	**0.786**
NCAP_CVHSA_0393-0407	SARS-CoV	N	VTLLPAADMDDFSRQ	$6.6X10^{-3}$	Mild/Sev	0.787
SPIKE_CVHSA_0185-0199	SARS-CoV	S	FVFKNKDGFLYVYKG	$4.8X10^{-3}$	Mild/Sev	0.926
SPIKE_CVHSA_0645-0659	SARS-CoV	S	SYECDIPIGAGICAS	$7.5X10^{-3}$	Mild/Sev	0.782
SPIKE_CVHSA_1129-1143	SARS-CoV	**S**	SFKEELDKYFKNHTS	**$2X10^{-3}$**	**Mild/Sev**	**0.822**
SPIKE_CVEMC_0109-0123	MERS	S	VKQFANGFVVRIGAA	$8X10^{-4}$	Mild/Sev	no
SPIKE_CVEMC_1225-1239	MERS	S	NSTGIDFQDELDEFF	$1.6X10^{-3}$	Asym/ Mild	0.963
SPIKE_CVEMC_1229-1243	MERS	S	IDFQDELDEFFKNVS	$8X10^{-4}$<br>$9.9X10^{-3}$	Asym/mild<br>Mild/Sev	0.981<br>0.773
SPIKE_CVH22_1133-1147	229E	**S**	LLLCCCSTGCCGFFS	**$2X10^{-3}$**	**Mild/Sev**	**0.822**
SPIKE_CVHNL_0557-0571	NL63	S	LNNFQKFKTICFSTV	$4.9X10^{-3}$	Mild/Sev	0.204
SPIKE_CVHOC_0757-0771	OC43	S	RGAITTGYRFTNFEP	$7X10^{-4}$	Mild/Sev	0.151
SPIKE_CVHN1_0759_0773	HKU1	S	RRSISASYRFVTFEP	$6X10^{-4}$	Mild/Sev	0.147
SPIKE_CVHN2_0757_0771	HKU1	S	GISSPYRFVTFEPFN	$6.6X10^{-3}$	Mild/Sev	0.213

Table 2S supplementary. Immunodominant epitopes for children group (comparison between groups pediatric/mild/severe)

Peptide	Organism	Protein	Sequence	p-value	Comparison Group	AUC
NCAP_CVEMC_0089-0103	MERS	N	NGIKQLAPRWYFYYT	$2.7X10^{-3}$	PED/MILD	0.18
NCAP_CVEMC_0093-0107	MERS	N	QLAPRWYFYYTGTGP	$1X10^{-4}$	PED/MILD	0.1
NCAP_CVEMC_0321-0335	MERS	N	DHGNPVYFLRYSGAI	$5.9X10^{-3}$<br>$9X10^{-4}$	PED/MILD<br>PED/SEVE	0.2<br>0.15
NCAP_CVEMC_0357-0371	MERS	N	AYKTFPKKEKKQKAP	$3.2X10^{-3}$	PED/MILD	**0.8**
SPIKE_CVEMC_0265-0279	MERS	S	LFSSRYVDLYGGNMF	$3X10^{-4}$	PED/MILD	0.13
SPIKE_CVEMC_0289-0303	MERS	S	TIKYYSIIPHSIRSI	$6.8X10^{-3}$	PED/SEVE	0.21
SPIKE_CVEMC_0493-0507	MERS	S	KPLKYSYINKCSRFL	$3X10^{-4}$	PED/MILD	0.13
SPIKE_CVEMC_1133-1147	MERS	S	GLYFMHVGYYPSNHI	$3.2X10^{-3}$	PED/MILD	0.18
NCAP_CVH22_0053-0067	229E	N	KLIGYWNVQKRFRTR	$1.9X10^{-3}$<br>$1.3X10^{-5}$	PED/MILD<br>PED/SEVE	0.17<br>0.067
NCAP_CVH22_0057-0071	229E	N	YWNVQKRFRTRKGKR	$6X10^{-4}$	PED/MILD	0.14
NCAP_CVH22_0189-0203	229E	N	ALKSLGFDKPQEKDK	$4.3X10^{-3}$	PED/MILD	**0.8**
SPIKE_CVH22_0189-0203	229E	S	TVREFVISRTGHFYI	$3.2X10^{-3}$	PED/SEVE	0.18
SPIKE_CVH22_0193-0207	229E	S	FVISRTGHFYINGYR	$8X10^{-4}$<br>$5.1X10^{-3}$	PED/MILD<br>PED/SEVE	0.14<br>0.2
SPIKE_CVH22_0197-0211	229E	S	RTGHFYINGYRYFTL	$1.1X10^{-3}$<br>$7.9X10^{-3}$	PED/MILD<br>PED/SEVE	0.15<br>0.2
SPIKE_CVH22_0201-0215	229E	S	FYINGYRYFTLGNVE	$4.3X10^{-3}$	PED/MILD	0.19
SPIKE_CVH22_0489-0503	229E	S	GTIYSITPCNPPDQL	$1.3X10^{-3}$	PED/MILD	**0.838**
SPIKE_CVH22_0989-1003	229E	S	YYRITSRIMFEPRIP	$1.1X10^{-3}$<br>$4.3X10^{-3}$	PED/MILD<br>PED/SEVE	0.15<br>0.19
SPIKE_CVH22_1097-1111	229E	S	STLVDLKWLNRVETY	$3.2X10^{-3}$	PED/MILD	0.18
SPIKE_CVH22_1105-1119	229E	S	LNRVETYIKWPWWVW	$3X10^{-4}$	PED/MILD	0.12
SPIKE_CVH22_1133-1147	229E	**S**	LLLCCCSTGCCGFFS	**$2X10^{-4}$**	**PED/MILD**	**0.114**
VME1_CVH22_0033-0047	229E	M	QFGHYKYSRLFYGLK	$1.9X10^{-3}$<br>$9.1X10^{-3}$	PED/MILD<br>PED/SEVE	0.17<br>0.2
VME1_CVH22_0101-0115	229E	M	LFRRARTFWAWNPEV	$1X10^{-4}$<br>$5X10^{-4}$	PED/MILD<br>PED/SEVE	0.09<br>0.1

NCAP_CVHNL_0077-0091	NL63	N	HFYYLGTGPHKDLKF	6.8X10^{-3}	PED/MILD	0.21
SPIKE_CVHNL_0101-0115	NL63	S	VTLKICKFSRNTTFD	5.9X10^{-3}	PED/SEVE	0.205
SPIKE_CVHNL_0149-0163	NL63	S	VRLHLYNVTRTFYVP	2.3X10^{-3}	PED/SEVE	0.176
SPIKE_CVHNL_0153-0167	NL63	S	LYNVTRTFYVPAAYK	5.1X10^{-3}<br>5.9X10^{-3}	PED/MILD<br>PED/SEVE	0.2<br>0.205
SPIKE_CVHNL_0541-0555	NL63	S	WHIYLKSGTCPFSFS	1X10^{-4}	PED/MILD	0.105
SPIKE_CVHNL_1285-1299	NL63	S	LLNRFENYIKWPWWV	1X10^{-4}	PED/MILD	0.11
VME1_CVHNL_0033-0047	NL63	M	LQYGHYKYSRLLYGL	3X10^{-4}<br>6.8X10^{-3}	PED/MILD<br>PED/SEVE	0.12<br>0.2
VME1_CVHNL_0193-0207	NL63	M	TGWAFYVRAKHGDFS	4.3X10^{-3}<br>3.2X10^{-3}	PED/MILD<br>PED/SEVE	0.19<br>0.18
NCAP_CVHOC_0113-0127	OC43	N	DGNQRQLLPRWYFYY	8X10^{-4}<br>5.9X10^{-3}	PED/MILD<br>PED/SEVE	0.14<br>0.2
NCAP_CVHOC_0121-0135	OC43	N	PRWYFYYLGTGPHAK	2.7X10^{-3}	PED/MILD	0.18
NCAP_CVHOC_0261-0275	OC43	N	EVRQKILNKPRQKRS	2X10^{-4}	PED/MILD	0.12
SPIKE_CVHOC_0081-0095	OC43	S	KGSVLLSRLWFKPPF	5X10^{-4}	PED/MILD	0.138
SPIKE_CVHOC_0085-0099	OC43	S	LLSRLWFKPPFLSDF	1.8X10^{-5}	PED/MILD	0.071
SPIKE_CVHOC_0089-0103	OC43	S	LWFKPPFLSDFINGI	1X10^{-4}	PED/MILD	0.095
SPIKE_CVHOC_0237-0251	OC43	S	LFNVYLGMALSHYYV	8X10^{-4}	PED/MILD	0.148
SPIKE_CVHOC_0261-0275	OC43	S	TLEYWVTPLTSRQYL	2.7X10^{-3}	PED/MILD	0.181
SPIKE_CVHOC_0445-0459	OC43	S	SRFNPSTWNKRFGFI	1.3X10^{-5}	PED/MILD	0.067
SPIKE_CVHOC_0561-0575	OC43	S	YCGGNSCTCRPQAFL	3X10^{-4}	PED/SEVE	0.124
SPIKE_CVHOC_1285-1299	OC43	S	KDIGTYEYYVKWPWY	2.7X10^{-3}	PED/MILD	0.181
NCAP_CVHSA_0101-0115	SARS-CoV	N	KMKELSPRWYFYYLG	5.9X10^{-3}	PED/MILD	0.2
NCAP_CVHSA_0157-0171	SARS-CoV	**N**	ATVLQLPQGTTLPKG	**6.8X10^{-3}**	**PEDI/MILD**	**0.79**
NCAP_CVHSA_0221-0235	SARS-CoV	N	ALLLLDRLNQLESKV	7.9X10^{-3}	PEDI/SEVER	**0.786**
NCAP_CVHSA_0253-0267	SARS-CoV	N	AEASKKPRQKRTATK	5X10^{-4}	PED/SEVE	0.13
SPIKE_CVHSA_0189-0203	SARS-CoV	S	NKDGFLYVYKGYQPI	2.7X10^{-3}	PED/MILD	0.181
SPIKE_CVHSA_0305-0319	SARS-CoV	S	FRVVPSGDVVRFPNI	1X10^{-4}	PED/MILD	0.1
SPIKE_CVHSA_0353-0367	SARS-CoV	S	SVLYNSTFFSTFKCY	7.9X10^{-3}	PED/MILD	0.214

SPIKE_CVHSA_0537-0551	SARS-CoV	S	**VLTPSSKRFQPFQQF**	$1.2X10^{-6}$<br>$2.7X10^{-3}$	PED/MILD<br>PED/SEVE	0.033<br>0.181
SPIKE_CVHSA_0541-0555	SARS-CoV	S	**SSKRFQPFQQFGRDV**	$3.7X10^{-3}$	PED/MILD	0.19
SPIKE_CVHSA_0833-0847	SARS-CoV	S	**CAQKFNGLTVLPPLL**	$1.3X10^{-3}$	PED/SEVE	**0.838**
SPIKE_CVHSA_1065-1079	SARS-CoV	S	**HEGKAYFPREGVFVF**	$3.2X10^{-3}$	PED/MILD	0.186
SPIKE_CVHSA_1125-1139	SARS-CoV	S	**PELDSFKEELDKYFK**	$1.3X10^{-3}$	PED/SEVE	**0.838**
SPIKE_CVHSA_1129-1143	SARS-CoV	S	**SFKEELDKYFKNHTS**	$2.7X10^{-3}$	PED/SEVE	**0.819**
VEMP_CVHSA_0061-0075	SARS-CoV	E	**RVKNLNSSEGVPDLL**	$5.1X10^{-3}$	PED/MILD	0.8
NCAP_SARS2_0109-0123	SARS-CoV-2	N	**YFYYLGTGPEAGLPY**	$9.1X10^{-3}$	PED/MILD	0.2
NCAP_SARS2_0221-0235	SARS-CoV-2	**N**	**LLLLDRLNQLESKMS**	**$8X10^{-4}$**	**PED/SEVE**	**0.852**
NCAP_SARS2_0393-0404	SARS-CoV-2	**N**	**TLLPAADLDDFSKQL**	**$1.9X10^{-3}$**	**PED/SEVE**	**0.829**
NS7A_SARS2_0009-0023	SARS-CoV-2	NS7A	**LITLATCELYHYQEC**	$3.2X10^{-3}$	PED/MILD	0.18
NS8_SARS2_0033-0047	SARS-CoV-2	NS8A	**VDDPCPIHFYSKWYI**	$5.9X10^{-3}$	PED/MILD	0.2
R1A_R1AB_SARS2_2001-2015	SARS-CoV-2	R<br>(Nsp)	**ATYKPNTWCIRCLWS**	$3X10^{-4}$	PED/MILD	0.12
R1A_R1AB_SARS2_2157-2171	SARS-CoV-2	R<br>(Nsp)	**VTRCLNRVCTNYMPY**	$1.3X10^{-5}$<br>$4.3X10^{-3}$	PED/MILD<br>PED/SEVE	0.07<br>0.2
R1A_R1AB_SARS2_3005-3019	SARS-CoV-2	R<br>(Nsp)	**VLNNDYYRSLPGVFC**	$5X10^{-4}$	PED/MILD	0.13
R1A_R1AB_SARS2_3469-3483	SARS-CoV-2	R<br>(Nsp)	**AWLYAAVINGDRWFL**	$5.1X10^{-3}$	PED/MILD	0.2
R1A_R1AB_SARS2_4269-4283	SARS-CoV-2	R<br>(Nsp)	**FCAFAVDAAKAYKDY**	$3.7X10^{-3}$	PED/MILD	0.19
R1AB_SARS2_4541-4555	SARS-CoV-2	R<br>(Nsp)	**YNCCDDDYFNKKDWY**	$9.1X10^{-3}$	PED/MILD	0.2
R1AB_SARS2_4653-4667	SARS-CoV-2	R<br>(Nsp)	**LTKPYIKWDLLKYDF**	$1X10^{-4}$	PED/MILD	0.1
R1AB_SARS2_4673-4687	SARS-CoV-2	R<br>(Nsp)	**KLFDRYFKYWDQTYH**	$1.6X10^{-3}$	PED/MILD	0.16
R1AB_SARS2_4725-4739	SARS-CoV-2	R<br>(Nsp)	**IFVDGVPFVVSTGYH**	$6.8X10^{-3}$	PED/MILD	0.2
R1AB_SARS2_4729-4743	SARS-CoV-2	R<br>(Nsp)	**GVPFVVSTGYHFREL**	$7.9X10^{-3}$<br>$1.6X10^{-3}$	PED/MILD<br>PED/SEVE	0.2<br>0.16
R1AB_SARS2_4809-4823	SARS-CoV-2	R<br>(Nsp)	**KDFYDFAVSKGFFKE**	$5.9X10^{-3}$	PED/MILD	0.2

R1AB_SARS2_4845-4859	SARS-CoV-2	R (Nsp)	**YDYYRYNLPTMCDIR**	$6X10^{-4}$	PED/MILD	0.14
R1AB_SARS2_5273-5287	SARS-CoV-2	R (Nsp)	**FHLYLQYIRKLHDEL**	$5X10^{-4}$	PED/MILD	0.13
R1AB_SARS2_5829-5843	SARS-CoV-2	R (Nsp)	**AWRKAVFISPYNSQN**	$6.8X10^{-3}$	PED/SEVE	0.27
R1AB_SARS2_6073-6087	SARS-CoV-2	**R (Nsp)**	**HLIPLMYKGLPWNVV**	**$4X10^{-4}$**	**PED/MILD**	**0.133**
R1AB_SARS2_6153-6167	SARS-CoV-2	R (Nsp)	**HHSIGFDYVYNPFMI**	$2.7X10^{-3}$	PED/MILD	0.18
R1AB_SARS2_6681-6695	SARS-CoV-2	R (Nsp)	**GYAFEHIVYGDFSHS**	$7.9X10^{-3}$	PED/MILD	0.2
R1AB_SARS2_6973-6987	SARS-CoV-2	R (Nsp)	**SWNADLYKLMGHFAW**	$1.3X10^{-3}$	PED/MILD	0.16
SPIKE_SARS2_0133-0147	SARS-CoV-2	S	**FQFCNDPFLGVYYHK**	$8X10^{-4}$	PED/MILD	0.148
SPIKE_SARS2_0265-0279	SARS-CoV-2	S	**YYVGYLQPRTFLLKY**	$7.9X10^{-3}$	PED/SEVE	0.214
SPIKE_SARS2_0325-0339	SARS-CoV-2	S	**SIVRFPNITNLCPFG**	$1.6X10^{-3}$<br>$2.7X10^{-3}$	PED/MILD<br>PED/SEVE	0.167<br>0.181
SPIKE_SARS2_0553-0567	SARS-CoV-2	S	**TESNKKFLPFQQFGR**	$3.7X10^{-3}$	PED/MILD	0.19
SPIKE_SARS2_0557-0571	SARS-CoV-2	**S**	**KKFLPFQQFGRDIAD**	**$3.2X10^{-3}$**	**PED/MILD**	**0.186**
SPIKE_SARS2_0785-0799	SARS-CoV-2	**S**	**VKQIYKTPPIKDFGG**	**$2.7X10^{-3}$**	**PED/MILD**	**0.181**
SPIKE_SARS2_1145-1159	SARS-CoV-2	**S**	**LDSFKEELDKYFKNH**	**$3.7X10^{-3}$**	**PED/SEVE**	**0.81**
SPIKE_SARS2_1201-1215	SARS-CoV-2	S	**QELGKYEQYIKWPWY**	$9X10^{-4}$	PED/MILD	0.15
SPIKE_SARS2_1205-1219	SARS-CoV-2	S	**KYEQYIKWPWYIWLG**	$3.7X10^{-3}$	PED/MILD	0.19
VME1_SARS2_0005-0019	SARS-CoV-2	M	**NGTITVEELKKLLEQ**	$7.9X10^{-3}$	PED/MILD	0.2
SPIKE_CVHN1_0452_0466	HKU1	S	**NFNLSSHSVVYSRYC**	$1.9X10^{-3}$	PED/MILD	0.171
SPIKE_CVHN1_1144_1158	HKU1	S	**GLLFMHFSYKPISFK**	$2.3X10^{-3}$<br>$3.2X10^{-3}$	PED/MILD<br>PED/SEVE	0.176<br>0.186
SPIKE_CVHN1_1176_1190	HKU1	S	**PKQGYFIKHNDHWMF**	$1X10^{-4}$	PED/MILD	0.11
SPIKE_CVHN2_0084_0098	HKU1	S	**YLSTLWYKPPFLSDF**	$1.8X10^{-3}$	PED/MILD	0.157
SPIKE_CVHN2_0088_0102	HKU1	S	**LWYKPPFLSDFNNGI**	$3.7X10^{-3}$	PED/MILD	0.19
VME1_CVHN1_0001_0015	HKU1	M	**MNKSFLPQFTSDQAV**	$5X10^{-4}$<br>$9X10^{-4}$	PED/MILD<br>PED/SEVE	**0.862**<br>**0.848**
