# Supplementary material for: Immune profiling of SARS-CoV-2 epitopes in asymptomatic and symptomatic pediatric and adult patients
Source: J Transl Med. 2023 Feb 14;21:123. doi: 10.1186/s12967-023-03963-5 (PMC9927035; doi:10.1186/s12967-023-03963-5)
Supplement: Supplementary file 3 — Additional file 3: Table S3. Percentage of individuals who developed IgG response against N and S proteins of the seven human coronaviruses in each patient group. [file 12967_2023_3963_MOESM3_ESM.pdf]

Viruses	PDC n=14 (%)	INT-A n=14 (%)	HOS n=16 (%)
MERS (CVEMC)	S (100)<br>N (71.4)	S (78.6)<br>N (85.7)	S (100)<br>N (87.5)
CVH22 (229E)	S (100)<br>N (64.3)	S (92.8)<br>N (64.2)	S (100)<br>N (68.7)
CVHN1 (HKU1)	S (85.7)<br>N (35.7)	S (78.6)<br>N (28.6)	S (68.7)<br>N (37.5)
CVHN2 (HKU1)	S (78.5)<br>N (7.1)	S (85.7)<br>N (28.5)	S (87.5)<br>N (31.2)
CVHN5 (HKU5)	S (78.6)<br>N (7.1)	S (85.7)<br>N (28.6)	S (87.5)<br>N (31.2)
CVHNL (NL63)	S (78.6)<br>N (35.7)	S (78.6)<br>N (50)	S (75)<br>N (31.2)
CVHOC (OC43)	S (100)<br>N (85.7)	S (100)<br>N (57.1)	S (100)<br>N (50)
CVHSA (SARS-CoV)	S (100)<br>N (85.7)	S (92.8)<br>N (71.4)	S (100)<br>N (100)
SARS2 (SARS-CoV-2)	S (78.5)<br>N (35.7)	S (64.3)<br>N (50)	S (93.7)<br>N (100)

Table 3. Percentage of individuals who developed IgG response against N and S proteins of the seven human coronaviruses in each patient group.
