# Supplementary material for: Immune profiling of SARS-CoV-2 epitopes in asymptomatic and symptomatic pediatric and adult patients
Source: J Transl Med. 2023 Feb 14;21:123. doi: 10.1186/s12967-023-03963-5 (PMC9927035; doi:10.1186/s12967-023-03963-5)
Supplement: Supplementary file 4 — Additional file 4: Table S4. Clinical features of hospitalized adult patients with SARS-CoV-2 infection. [file 12967_2023_3963_MOESM4_ESM.pdf]

Table 4S. Clinical features of hospitalized adult patients with SARS-CoV-2 infection

**Characteristic**	**Pre-intensive care** **n=3 (%)**	**Intensive care** **n=13 (%)**	**P value**
Glycaemia			**0.025**
≤99mg/dl	2 (66.6)	0	
>99mg/dl	1 (33.3)	13 (100)	
IL-6			0.486
≤100pg/ml	2 (13)	7 (47)	
>100pg/ml	0	6 (40)	
Azotemia			0.257
≤50mg/dl	1 (6.7)	1 (6.7)	
>50mg/dl	1 (6.7)	12 (80)	
Albumin			**0.007**
≤3.5g/dL	3 (100)	1 (7.7)	
>3.5g/dL	0	12 (92.3)	
Procalcitonin T0			0.529
≤0,5ng/ml	3 (100)	9 (69.2)	
>0,5ng/ml	0	4 (30.8)	
Prothrombin activity			
>28s	2 (66.6)	13 (100)	
D-Dimer			
>500ng/ml	0	9 (69.2)	
