# Supplementary material for: Immune profiling of SARS-CoV-2 epitopes in asymptomatic and symptomatic pediatric and adult patients
Source: J Transl Med. 2023 Feb 14;21:123. doi: 10.1186/s12967-023-03963-5 (PMC9927035; doi:10.1186/s12967-023-03963-5)
Supplement: Supplementary file 5 — Additional file 5: Table S5. Clinical and laboratory parameters of children hospitalized with SARS-CoV-2 infection. [file 12967_2023_3963_MOESM5_ESM.pdf]

Table 5S. Clinical and laboratory parameters of children hospitalized with SARS-CoV-2 infection

**Characteristic**	**Symptoms attributable to COVID 19** n=5	(%)	**Symptoms not attributable to COVID 19** n=14	(%)	**P value**
**Age**					0.405
0-7 years	4	(80)	8	(57.1)	
8-9 years	0		4	(28.6)	
10-12 years	1	(20)	2	(14.3)	
**Sex**					0.603
Male	2	(40)	9	(64.3)	
Female	3	(60)	5	(35.7)	
**Laboratory data**					
D-Dimer*					0.236
≤250ng/ml	0		4	(28.6)	
>250ng/ml	3	(60)	4	(28.6)	
Ferritin**					0.191
≤200ng/ml	3	(60)	11	(78.6)	
>200ng/ml	2	(40)	1	(7.2)	
Fibrinogen***					1.000
≤500ng/ml	3	(60)	8	(57.1)	
>500ng/ml	2	(40)	3	(21.4)	
PCR					0.141
<10mg/ml	1	(20)	9	(64.3)	
>10mg/ml	4	(80)	5	(35.7)	
PCT****					0.261
≤0.05ng/ml	0		4	(28.6)	
>0.05ng/ml	5	(100)	8	(57.1)	

PCR= Polymerase chain reaction; PCT= procalcitonin

*D-dimer testing was available for 11 children

**Ferritin testing was available for 17 children

***Fibrinogen testing was available for 16 children

****PCT testing was available for 17 children
