# Supplementary figures and images for: Immune profiling of SARS-CoV-2 epitopes in asymptomatic and symptomatic pediatric and adult patients
Source: J Transl Med. 2023 Feb 14;21:123. doi: 10.1186/s12967-023-03963-5 (PMC9927035; doi:10.1186/s12967-023-03963-5)

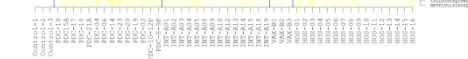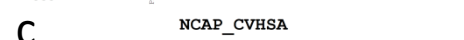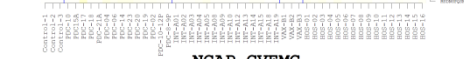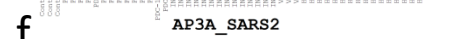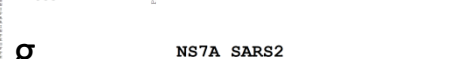

Supplement: Supplementary file 6 — Additional file 6: Fig. S1. Heatmaps obtained by probing the arrays with serum samples towards SARS-CoV-2 S (a), N (b), M (h), ORF3a (f), NS7 (g) and N proteins of CVHSA (SARS-CoV, c), CVEMC(MERS, d) and 229E (e). Each column represents the reaction of a serum sample against all viral epitopes. The rows represent overlapping peptides from the respective protein. Color codes indicate strong signals with red squares, low signals with yellow squares and no signal with white squares. Sample groups: 1) negative control obtained by probing the arrays with secondary antibody only, 2) Children samples (PDC), 3) adults samples with mild/moderate symptoms (INT-A) and vaccinated adults samples (VAX-B), 4) adults samples with severe symptoms (HOS). [file 12967_2023_3963_MOESM6_ESM.pdf]

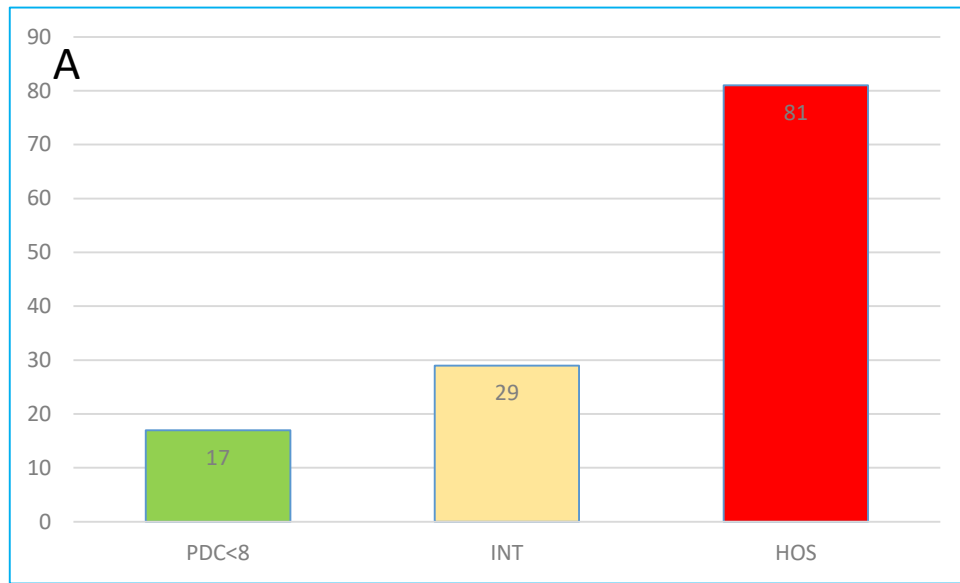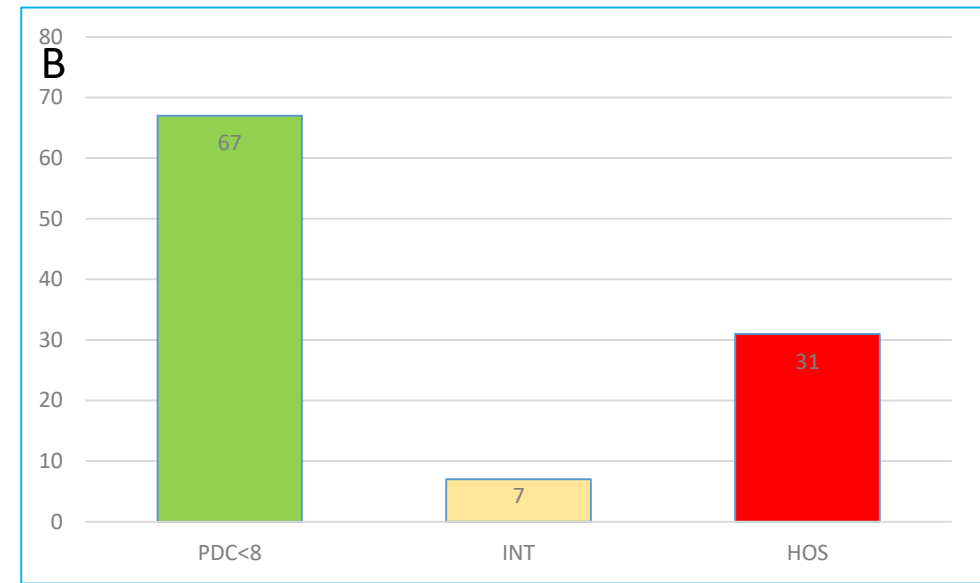

Supplement: Supplementary file 7 — Additional file 7: Fig.S2. A) Percentage of patients in each group, which reacted against peptides of S protein. B) Percentage of patients in each group, which reacted against peptides of M protein. [file 12967_2023_3963_MOESM7_ESM.pdf]

A)

Adults

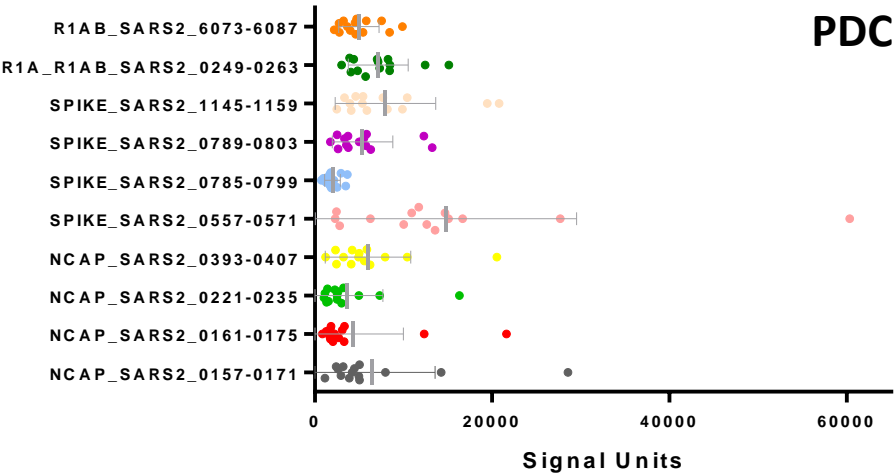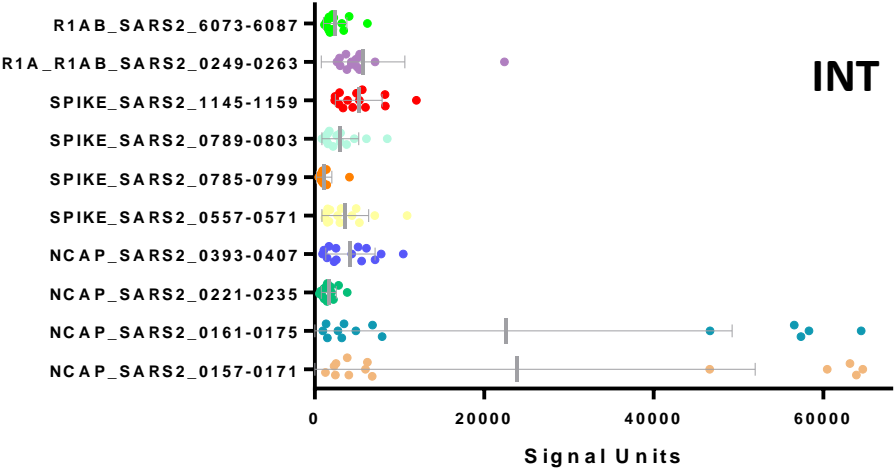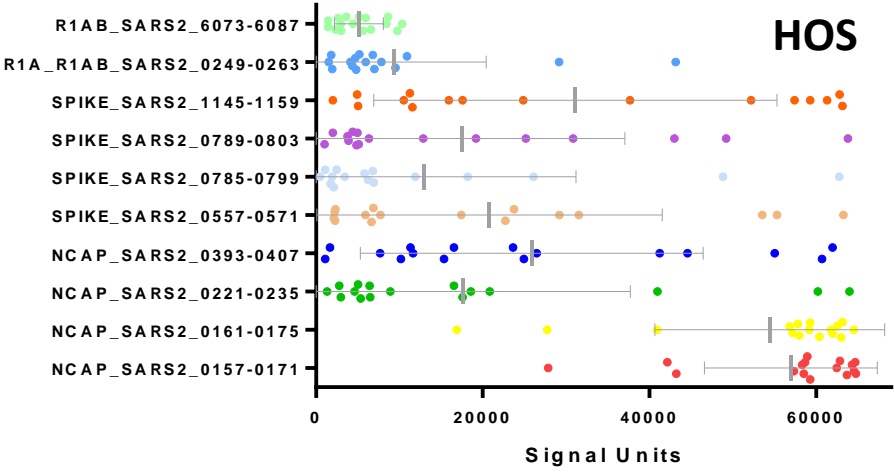

B)

Children

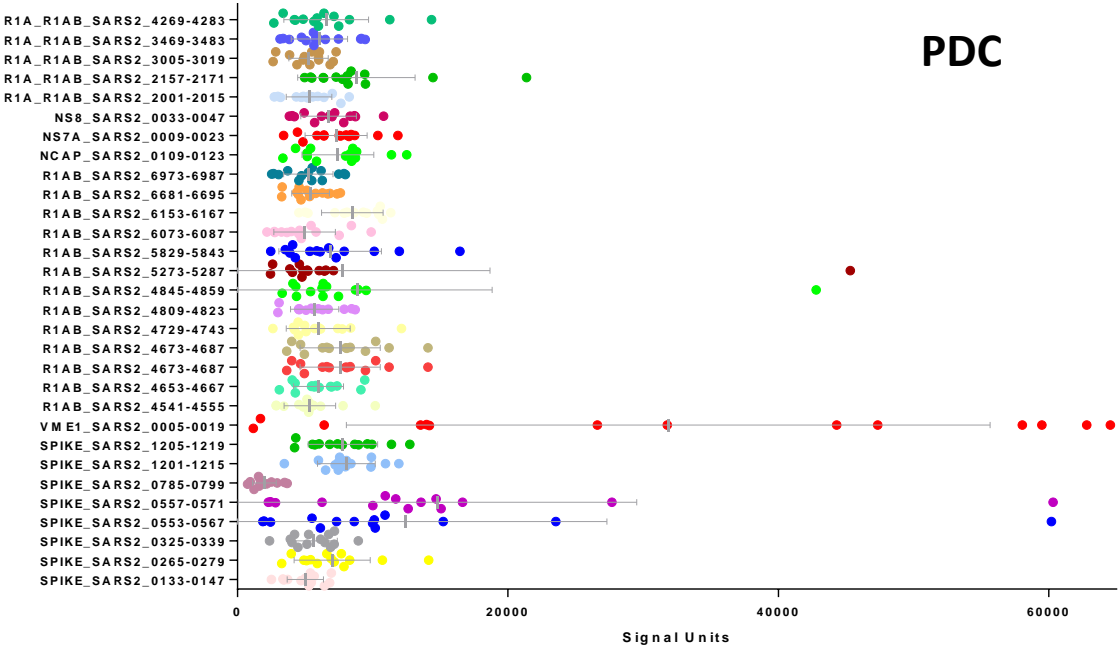

INT

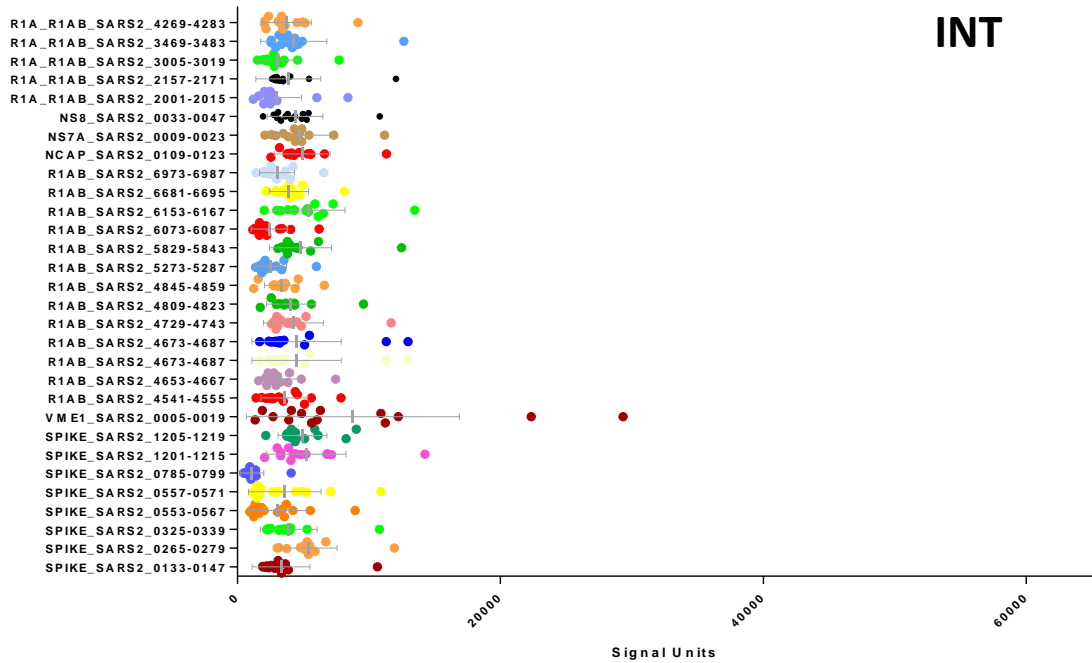

HOS

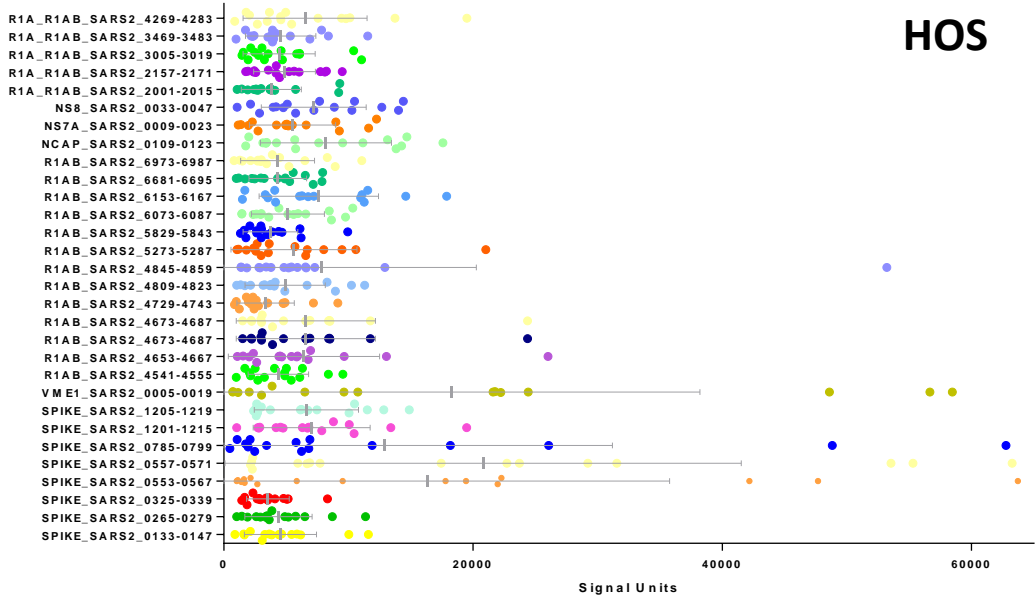

Supplement: Supplementary file 8 — Additional file 8: Fig. S3. Serum samples reactivity against SARS-CoV-2 epitopes: Samples from adults (A) and from children (B) with high reactivity towards epitopes of different proteins of SARS-CoV-2. [file 12967_2023_3963_MOESM8_ESM.pdf]

A

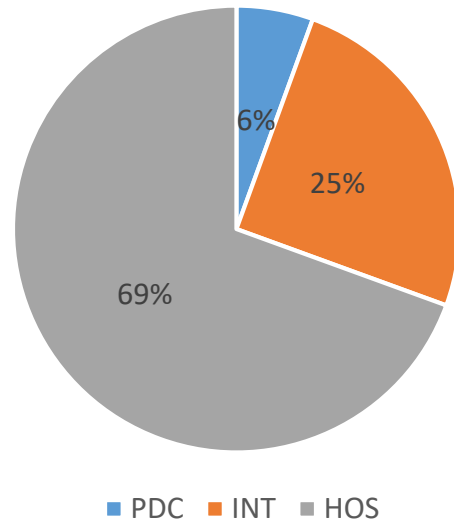

B

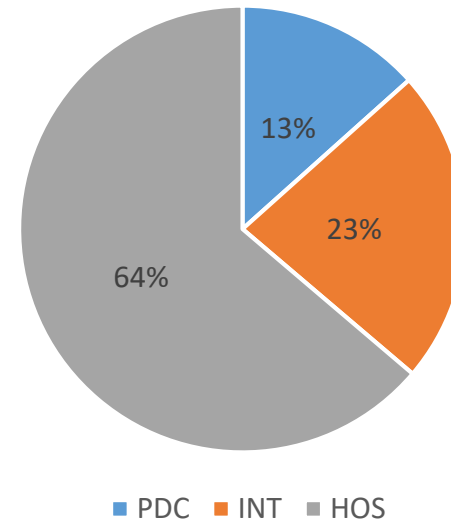

C

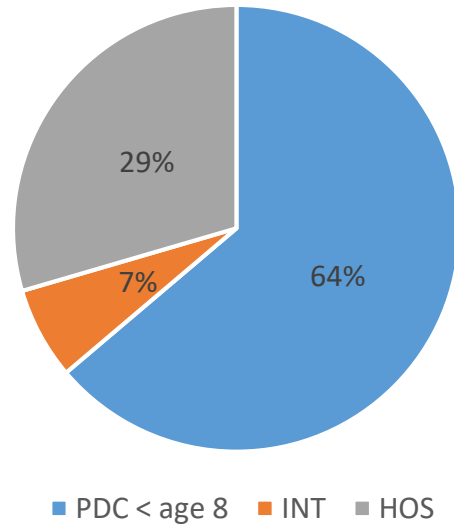

D

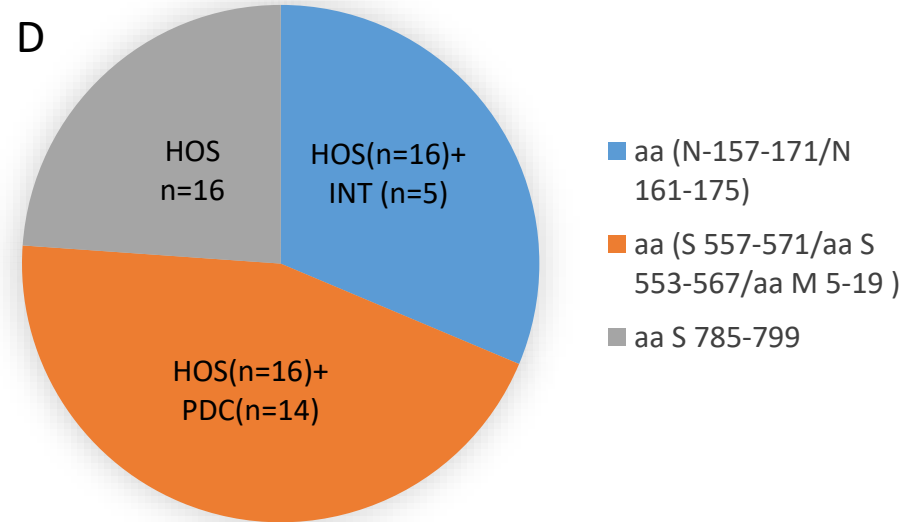

Supplement: Supplementary file 9 — Additional file 9: Fig.S4: A) Percentage of patients between groups who developed antibodies against epitopes of N protein, B) percentage of patients between groups who developed antibodies against epitopes of S protein; C) percentage of patients between group who developed antibodies against epitopes of M protein; D) Number of patients who developed antibodies against the most reactive epitopes. All HOS and 5 INT patients with moderate symptoms developed antibodies against two peptides from N protein (aa 157-171 and aa 161-175), all HOS and PDC developed antibodies against two peptides of S protein (aa 553-567 and aa 557-571) and against one peptide of M protein(aa 5-19); all HOS developed antibodies against the peptide aa 785-799 of S protein. [file 12967_2023_3963_MOESM9_ESM.pdf]

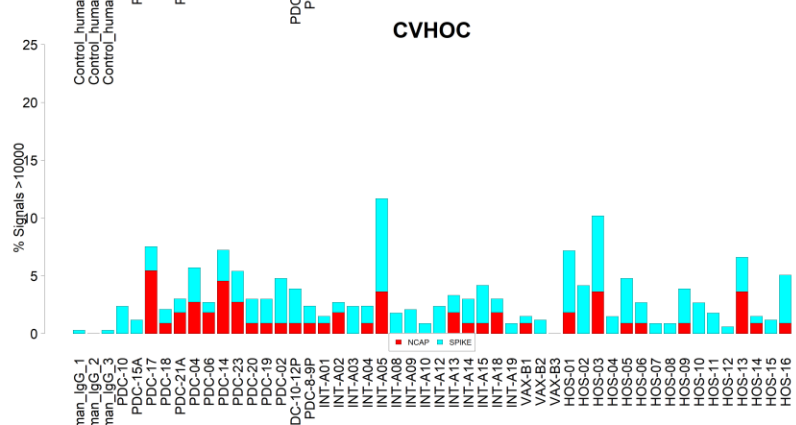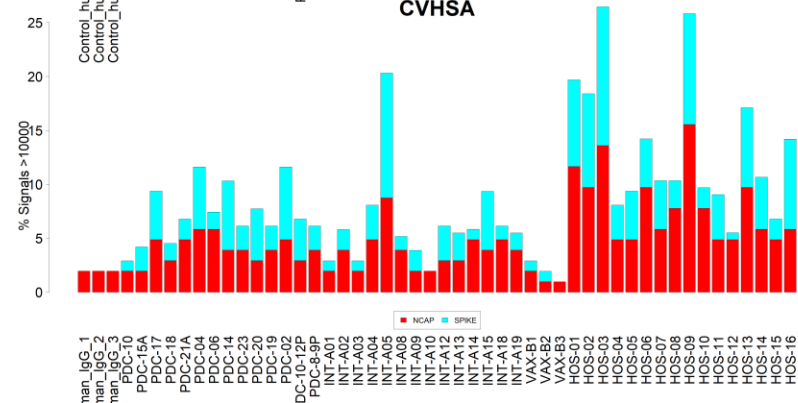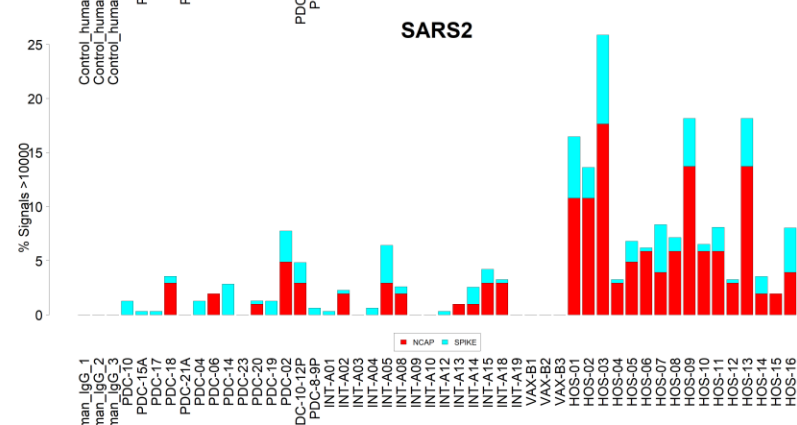

Supplement: Supplementary file 10 — Additional file 10: Fig. S5. Barplot showing the fraction of serum signals reacting towards N and S proteins of MERS and common-cold coronaviruses obtained by setting the threshold above 10,000. Control samples are reaction controls with the secondary antibody only (n=3), the PDC identifies samples from SARS-CoV-2 positive children, INT identifies samples from adults with mild/moderate symptoms and HOS the hospitalized patients with severe symptoms. Samples labeled as VAX-B1, VAX-B2 and VAX-B3 are each a pool of sera from 10 vaccinated subjects. [file 12967_2023_3963_MOESM10_ESM.pdf]

Adults

A)

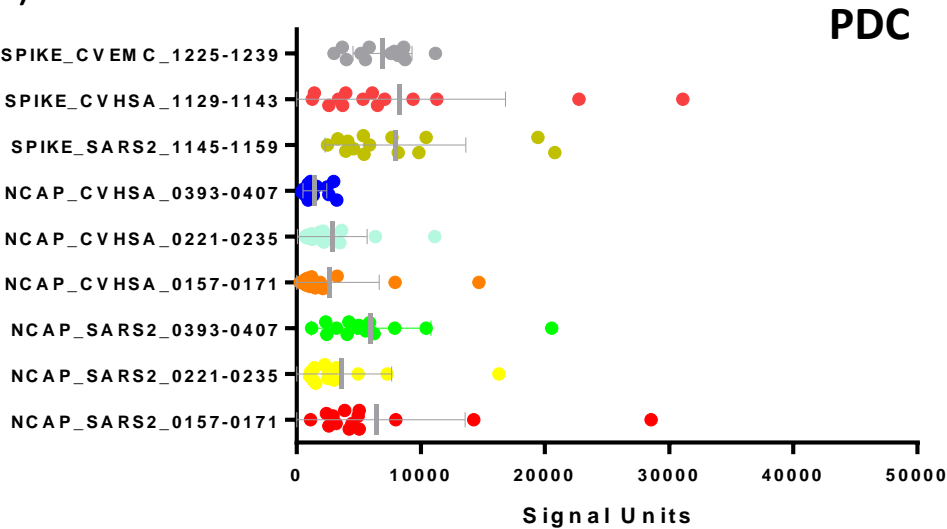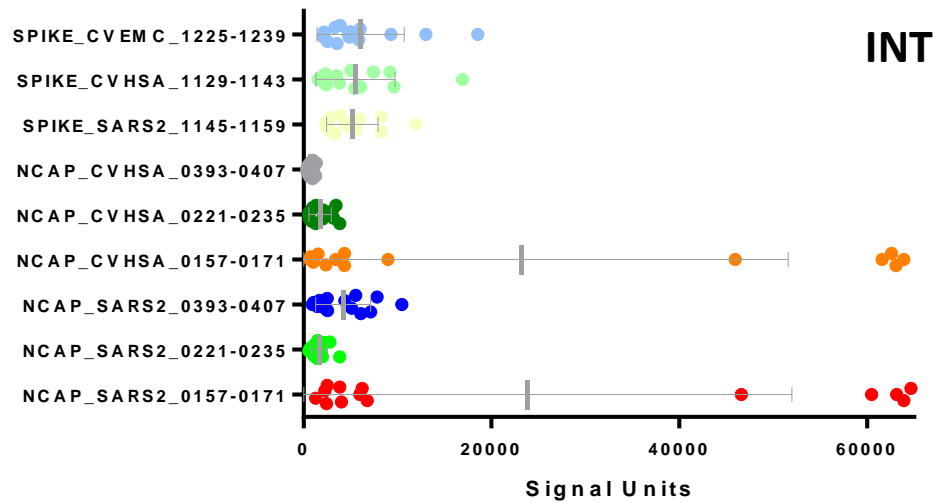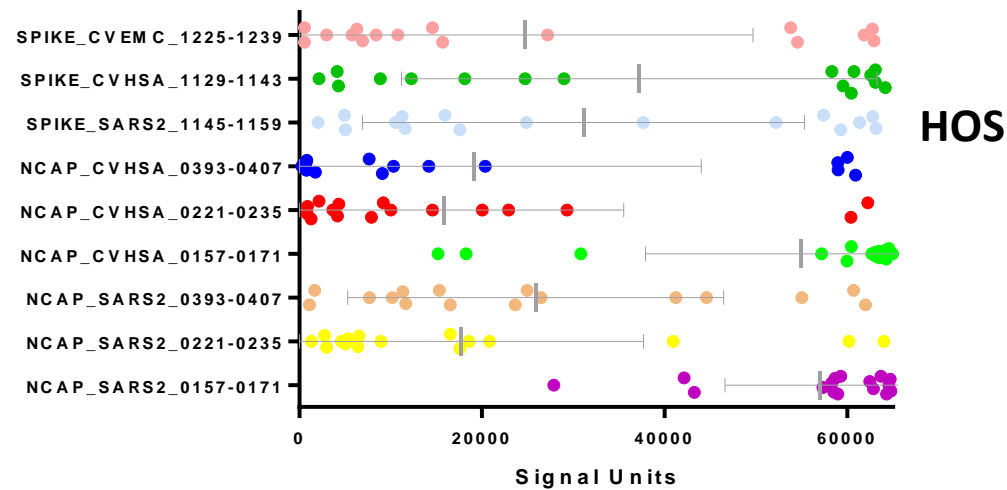

Children

B)

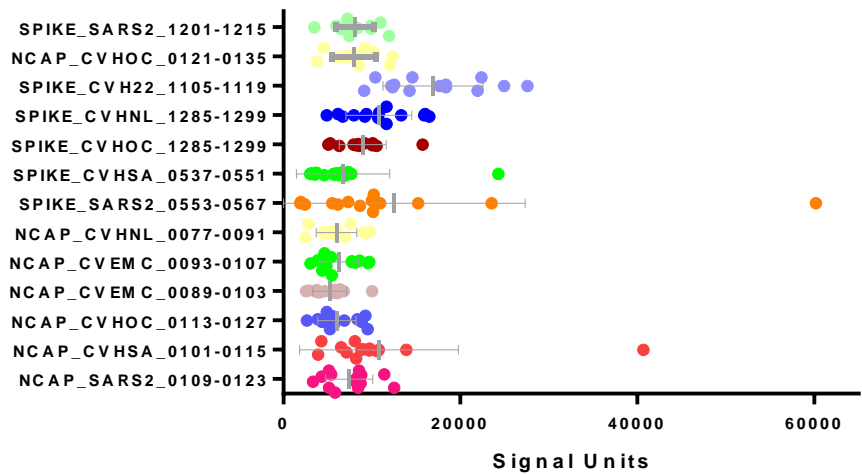

PDC

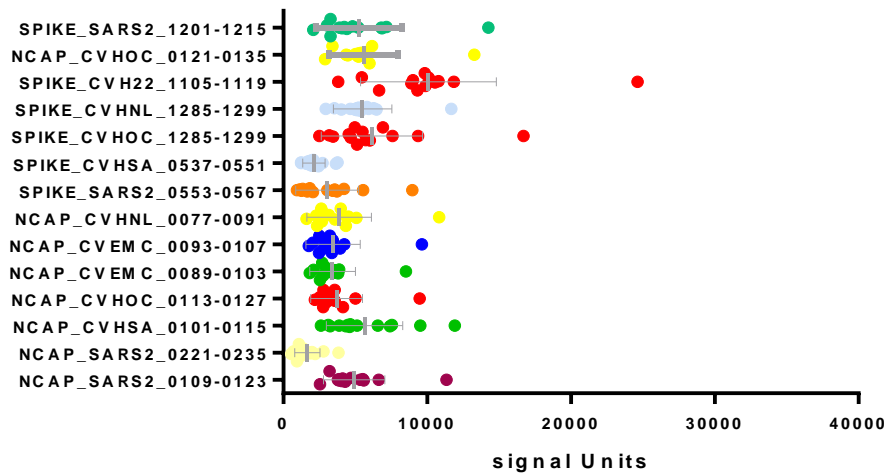

INT

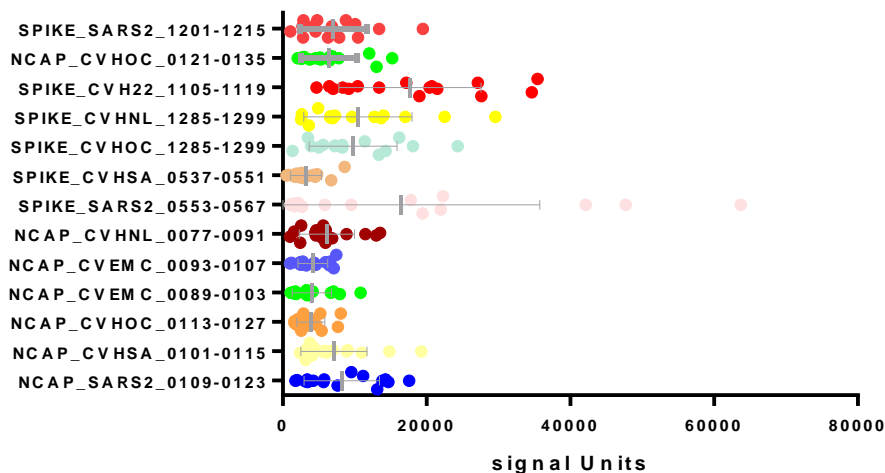

HOS

Supplement: Supplementary file 11 — Additional file 11: Fig. S6. Antibodies reactivity against MERS and common-cold coronaviruses epitopes: adult serum samples (A) and children serum samples (B) with high reactivity against epitopes of different coronaviruses. [file 12967_2023_3963_MOESM11_ESM.pdf]
